# Targeting soluble amyloid-beta oligomers with a novel nanobody

**DOI:** 10.1038/s41598-024-66970-6

**Published:** 2024-07-12

**Authors:** Justin R. Haynes, Clayton A. Whitmore, William J. Behof, Charlotte A. Landman, Henry H. Ong, Andrew P. Feld, Isabelle C. Suero, Celeste B. Greer, John C. Gore, Printha Wijesinghe, Joanne A. Matsubara, Brian E. Wadzinski, Benjamin W. Spiller, Wellington Pham

**Affiliations:** 1 Vanderbilt University Institute of Imaging Science, Vanderbilt University Medical Center, Nashville, TN 37232 USA; 2 Department of Radiology and Radiological Sciences, Vanderbilt University Medical Center, Nashville, TN 37232 USA; 3 Department of Biomedical Informatics, Vanderbilt University Medical Center, Nashville, TN 37232 USA; 4 Vanderbilt Brain Institute, Vanderbilt University, Nashville, TN 37232 USA; 5 Department of Ophthalmology and Visual Sciences, University of British Columbia, Vancouver, BC V5Z3N9 Canada; 6 Department of Pharmacology, Vanderbilt University, Nashville, TN 37232 USA; 7 Department of Biomedical Engineering, Vanderbilt University, Nashville, TN 37235 USA; 8 Vanderbilt Ingram Cancer Center, Nashville, TN 37232 USA; 9 Vanderbilt Institute of Chemical Biology, Vanderbilt University, Nashville, TN 37232 USA; 10 Vanderbilt Institute of Nanoscale Science and Engineering, Vanderbilt University, Nashville, TN 37235 USA; 11 Vanderbilt Center for Structural Biology, Vanderbilt University, Nashville, TN 37235 USA; 12 Vanderbilt Memory and Alzheimer’s Center, Vanderbilt University Medical Center, Nashville, TN 37212 USA

**Keywords:** Biotechnology, Chemical biology, Drug discovery, Neuroscience, Biomarkers

## Abstract

The classical amyloid cascade hypothesis postulates that the aggregation of amyloid plaques and the accumulation of intracellular hyperphosphorylated Tau tangles, together, lead to profound neuronal death. However, emerging research has demonstrated that soluble amyloid-β oligomers (SAβOs) accumulate early, prior to amyloid plaque formation. SAβOs induce memory impairment and disrupt cognitive function independent of amyloid-β plaques, and even in the absence of plaque formation. This work describes the development and characterization of a novel anti-SAβO (E3) nanobody generated from an alpaca immunized with SAβO. In-vitro assays and in-vivo studies using 5XFAD mice indicate that the fluorescein (FAM)-labeled E3 nanobody recognizes both SAβOs and amyloid-β plaques. The E3 nanobody traverses across the blood–brain barrier and binds to amyloid species in the brain of 5XFAD mice. Imaging of mouse brains reveals that SAβO and amyloid-β plaques are not only different in size, shape, and morphology, but also have a distinct spatial distribution in the brain. SAβOs are associated with neurons, while amyloid plaques reside in the extracellular matrix. The results of this study demonstrate that the SAβO nanobody can serve as a diagnostic agent with potential theragnostic applications in Alzheimer’s disease.

## Introduction

**Figure 1:**
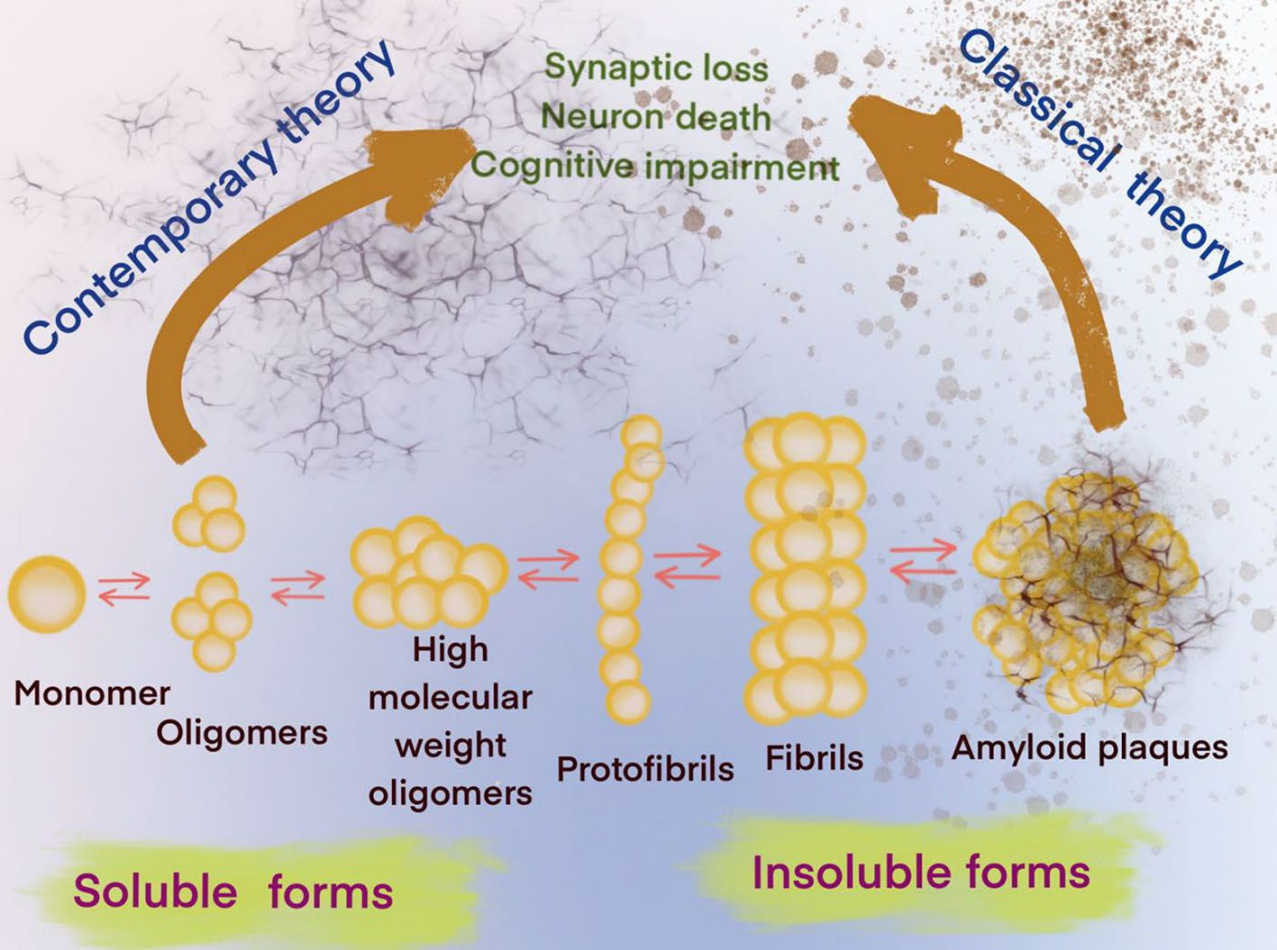
Contemporary and classical hypotheses of the amyloid-β cascade mechanisms.

In an aging population, AD will present one of the greatest challenges to medicine in this century. While the mechanisms underlying neuronal degeneration in AD remain elusive, the cytopathologic hallmarks of AD appear to be the formation of amyloid-β plaques between neurons and the intracellular accumulation of hyperphosphorylated Tau species. These processes are compounded by the reduction of amyloid-β clearance from the brain^1^, which ultimately leads to profound neuronal toxicity and tissue atrophy^2^. Originally, the amyloid cascade hypothesis considered soluble amyloid-β oligomers (SAβOs) as intermediates that would subsequently aggregate into protofibrils, insoluble fibrils and amyloid-β plaques, which were thought to be the main pathogenic cause of AD^3^ (Fig. 1). This hypothesis indicates that targeting SAβO could be a beneficial early AD intervention.

Experimental evidence ranging from cell studies to neuropathological and behavioral data suggests that elevated SAβO levels in the brain have pathological consequences^3^. As more data have emerged, it is now accepted that SAβOs exist for an extended periods without conversion to fibrillar isoforms^4^, and that SAβOs induce cognitive impairment in AD^5^. Since SAβOs are determinants of the severity of neurodegeneration in AD^6^, they could potentially serve as a biomarker for theragnostic applications^5,7–9^. SAβO is a synaptotoxin found at elevated levels and associated with synapses in the brains of AD patients, but not in healthy elderly people^10–12^. While several reports have implicated SAβOs as the cause of synaptic failure and neurodegeneration in AD^13–20^, more detailed studies suggest that SAβOs cause neuronal death due to perturbation of normal membrane functions^21^. SAβOs also induce memory impairment and disrupt cognitive function long before amyloid-β plaque deposition or even without plaque formation in a mouse model of AD^22–29^ (Fig. 1). Together, these findings indicate that SAβOs act upstream of amyloid-β plaques and Tau to promote neuronal dysfunction. Thus, reagents that target SAβOs are needed, for the prevention of other pathological events, such as activation of Tau- and amyloid-β plaque-dependent pathways that lead to an increase in severity of the disease.

Two types of approaches have been applied to target pathological amyloid proteins. One is the use of small organic molecules that mimick known amyloid-binding molecules^30,31^, and the other is the development and use of biologics^32–34^. Organic molecules that bind to β-sheets within the amyloid fibrils or plaques inhibit growth by blocking binding sites for additional Aβ molecules. The chemical backbone of these organic molecules is restricted to a few structures of the benzothiazoles or stilbene families. Many PET probes targeting amyloid fibrils/plaques were derived from these templates. For example, [^11^C]PIB and Flutemetamol are based on the benzothiazole structure, while Florbetapir and Florbetaben are derived from stilbenes. Because SAβOs lack the ordered beta solenoid structure of fibrils, developing small molecules to target SAβOs is more challenging.

In contrast, the development of biologics could serve as a promising strategy to target amyloid-β aggregates. Because SAβOs are made up of a few trimers or tetramers, they lack traditional binding surface for small molecules. Antibodies can recognize conformational epitopes unique to different oligomers. For instance, the mAb158 monoclonal antibody exhibits distinctive selectivity for soluble amyloid-β protofibrils compared to monomeric amyloid-β. This antibody binds preferentially to soluble protofibrils over mature, insoluble fibrils but has no affinity for amyloid precursor protein (APP)^35–38^. M94 is a polyclonal antibody that was purified from antisera of host animals immunized with synthetic amyloid-β oligomers. M94 shows high selectivity for pathogenic amyloid-β oligomers but not for physiological monomers^8^. Furthermore, ex-vivo staining of brain sections using the SAβO-specific antibodies revealed that SAβOs are distributed differently than neuritic plaques^39^. Overall, these studies highlight the prospect of biologics for targeting dementia-associated amyloid oligomers.

In this work, we focused on the development of a nanobody targeting SAβOs. We also describe assays to validate the nanobody binding specificity using ex-vivo and in-vivo models. Nanobodies (sometimes referred to as VHH) are derived from heavy chain-only IgG isotypes found in animals of the camelid family^40^. Nanobodies are approximately 1/10th the size of conventional antibodies (150 kDa). Despite their small size, nanobodies have unique specificities, potent affinities, and also exhibit high thermal stability and solubility^41^. Nanobodies also exhibit relatively short serum half-lives of approximately 2 h^42^, making them ideal for imaging applications. Additionally, nanobodies have been shown to cross biological barriers, including the blood–brain barrier (BBB)^43,44^, which makes them attractive for AD applications. Finally, the lack of antibody heavy chain constant regions would limit risk of Amyloid Related Imaging Abnormalities (ARIA)^45,46^. The results obtained from this work suggest that the E3 nanobody can recognize both SAβOs and amyloid-β plaques in-vivo. This probe enables us to observe the distinct spatial distribution of SAβO compared to amyloid-β plaques.

## Results

### Generation of SAβO as an antigen

**Figure 2:**
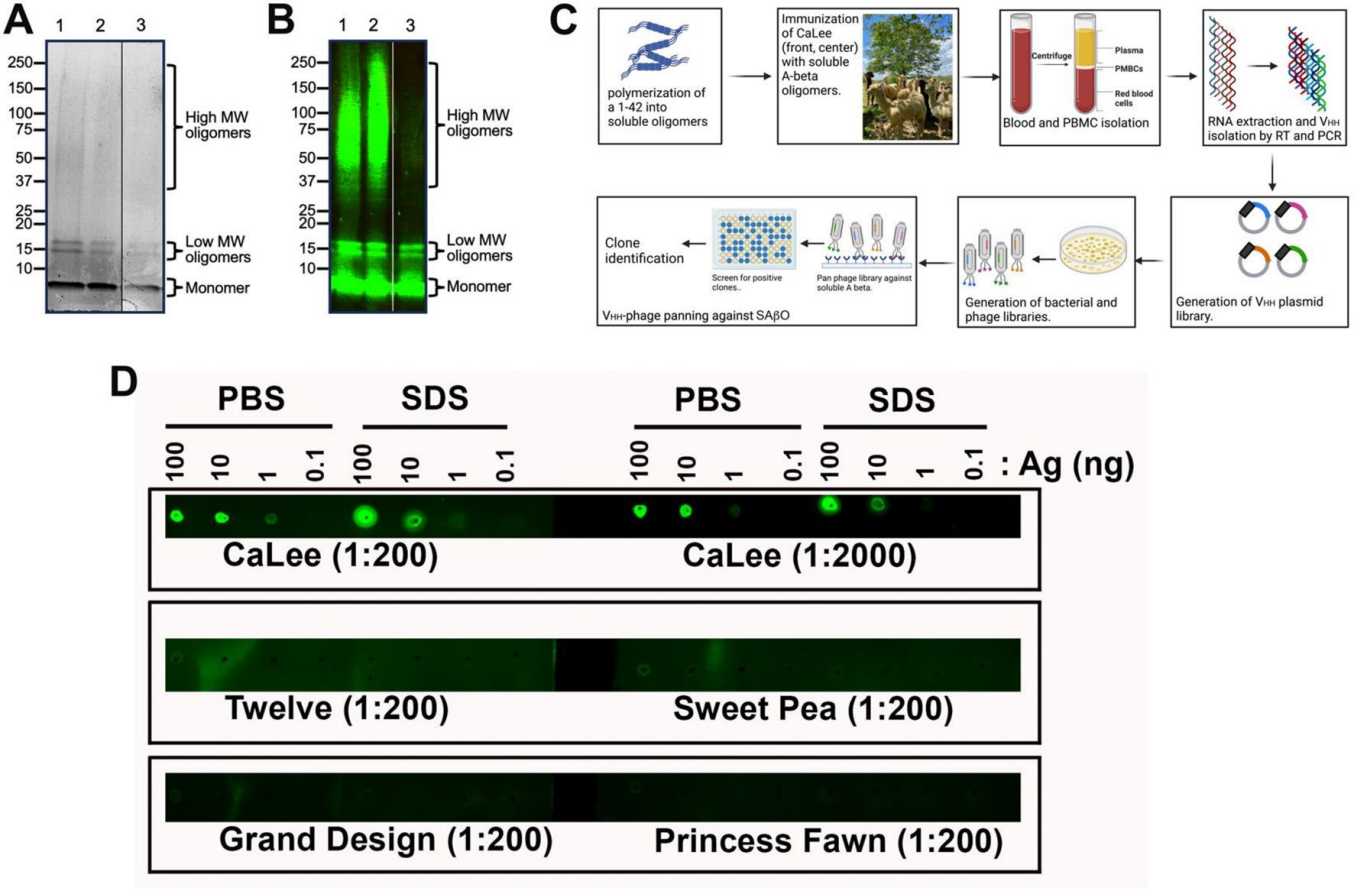
Production of nanobody from SAβO-injected alpaca. (**A**) Coomassie-stained SDS-polyacrylamide gel, and (**B**) Western analysis to confirm the production of the low molecular weight SAβOs (16 kDa) and some higher molecular species. Lanes 1 and 2 are from two different SAβO preparations; lane 3 is from free amyloid-β peptide. (**C**) General procedures for generating antibodies/nanobodies after immunization of alpaca with SAβO antigen. (**D**) Dot-blot analysis showed that the plasma from the alpaca immunized with SAβO (CaLee) recognizes SAβO antigen, while no response was observed for other non-immunized alpacas (Twelve, Sweety Pea, Grand Design, and Princess Fawn).

This work demonstrates the generation of batches of human SAβOs as an antigen for alpaca immunization. Based on prior work^47^, the inclusion of 0.05% SDS in the preparation helped to induce stable oligomers. After confirmation of the generation of SAβOs by Coomassie Blue gel staining, and the material was stored at − 20 °C before use. The assembled amyloid-β preparation contained three major components that included monomers, low molecular weight oligomers (~ 16 kDa), and high molecular weight oligomers (Fig. 2A,B; original blots shown as Figs. S1, S2 are available in the Supplementary Information). The monomer concentration is inversely proportional to assembly time. At the beginning of the seeding process, the monomer dominates the solution and migrates at 4–4.5 kDa. However, overtime, this band gets lighter, indicating the formation of higher-order oligomers at the expense of their monomer counterparts (data not shown). The low molecular weight oligomers are primarily composed of tetramers migrating at approximately 16 kDa. We also observed a small amount of higher molecular weight SAβO species in the 37–150 kDa range.

### Development and characterization of SAβO nanobodies

The overall procedure for generating anti-SAβO nanobodies is highlighted in Fig. 2C. We immunized an alpaca, CaLee, with SAβO antigen 7 times over the course of 84 days. Blood collections were performed 1 week after the 5th and 7th injections, and the plasma was tested for the presence of antibodies recognizing SAβO. The plasma obtained from CaLee, but not the control plasma from other alpacas, recognized the SAβO by dot-blot analysis (Fig. 2D). Nanobodies were isolated following an established protocol^48^. We generated a phage display library using cDNA derived from the PBMC mRNA and performed affinity panning of the phage library. Positive clones were identified by ELISA and clones identified multiple times were prioritized (Fig. S3, Supplementary Information). Six clones were tested in 8-point dilution series and positive clones were prioritized. From this operation, we identified the 6 best clones that bind SAβO (D11, E3, F9, B10, D4 and H5).

**Figure 3:**
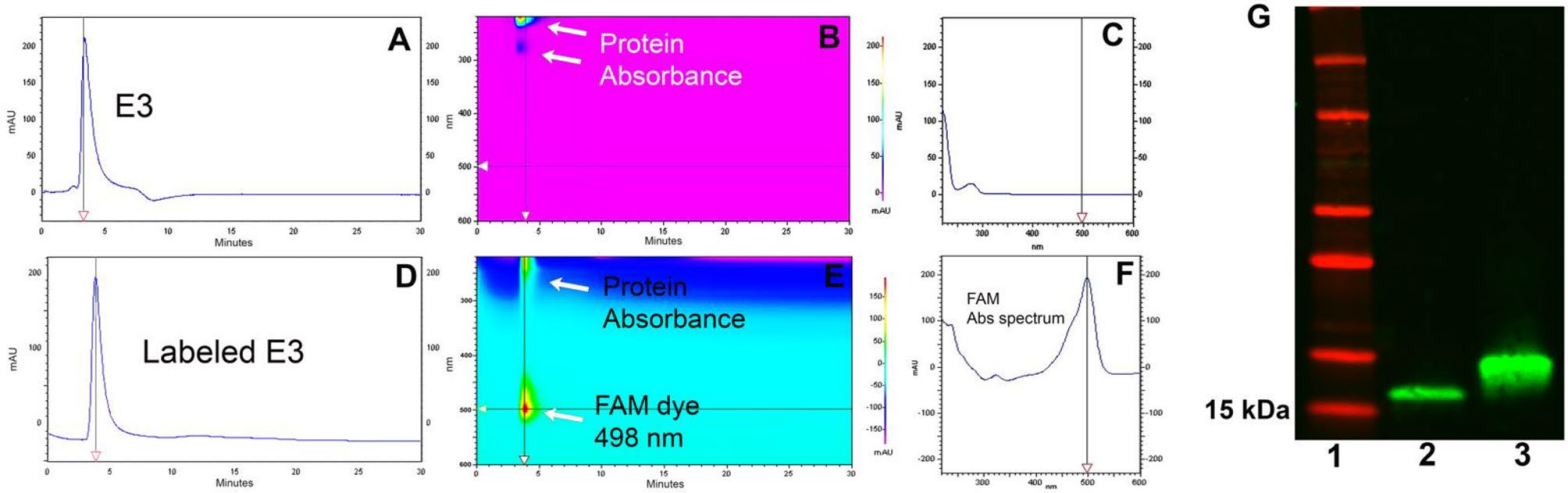
Representative data for the characterization of a dye-labeled clone. (**A**, **B**, **C**) Two-dimensional HPLC assay using a Cytiva HiTrap Sepharose column to characterize E3 nanobody and its labeled product. Unlabeled E3 nanobody. (**D**, **E**, **F**) FAM-labeled E3 nanobody. (**G**) Western blot analysis to show the purity of E3 (lane 2) and confirm FAM-labeled labeled E3 (lane 3). Molecular weight markers are shown in lane 1.

**Figure 4:**
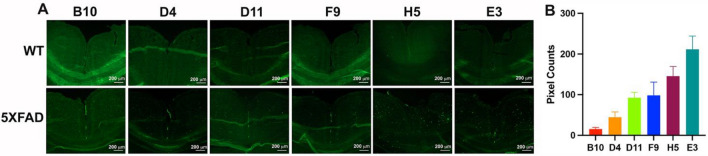
Screening for the specificity of the nanobody using 5XFAD mouse brain slides. (**A**) Ex-vivo evaluation of SAβO-binding specificity of the nanobody clones using consecutive brain slides obtained from a WT (6-month-old) and a 5XFAD mouse (11-month-old). (**B**) Ranking of nanobody clones after pixel quantification of 5XFAD brain slides. The threshold of each image was adjusted until the background disappeared using ImageJ. This process was repeated 3 times, and the data were presented as a mean ± SD.

We also developed an ex-vivo assay using mouse brain tissue sections mounted onto glass slides to screen for nanobody binding specificity. To facilitate the screening process, the nanobody clones were all labeled with fluorescein-NHS (FAM) dye. After removal of excess dye using a desalting spin column (Zepa columns, 7 K, MWCO, 2 mL), confirmation of the labeling was done via 2D-HPLC (Fig. 3A–F). As shown in the representative data of the E3 nanobody, the chromatogram profile of FAM-labeled E3 nanobody is nearly identical to that of the unlabeled counterpart, albeit with a longer retention time. Aside from having similar absorbance characteristic of a protein at 220–280 nm, the FAM-labeled product has an additional absorbance of FAM dye at 498 nm (Fig. 3E,F). Western analysis also confirmed the FAM-labeled E3 product (Fig. 3G; original data shown as Fig. S4 is available in the Supplementary Information). The FAM-labeled nanobody clones were used to stain 5XFAD and WT brain slides. To compare the binding specificity of the clones, we incubated FAM-labeled nanobodies with consecutive brain sections for 1 h at room temperature and imaged the slides using a fluorescent microscope (Fig. 4A). Full images of the screened clones can be found in the Supporting Information (Fig. S5). The screening data were exported to ImageJ to quantify the fluorescent signal and rank the nanobodies. Based on these data, we rank the nanobodies from best to worst: E3 > H5 > G11 = F9 > D4 > B10 (Fig. 4B). This ex-vivo staining process was repeated using two different 5XFAD brains, and the data confirmed E3 is the best among the clones. From here, we selected the E3 nanobody clone for further characterization.

### FAM-labeled E3 nanobody recognizes SAβOs and amyloid-β plaques in an ex-vivo staining of brain sections

**Figure 5:**
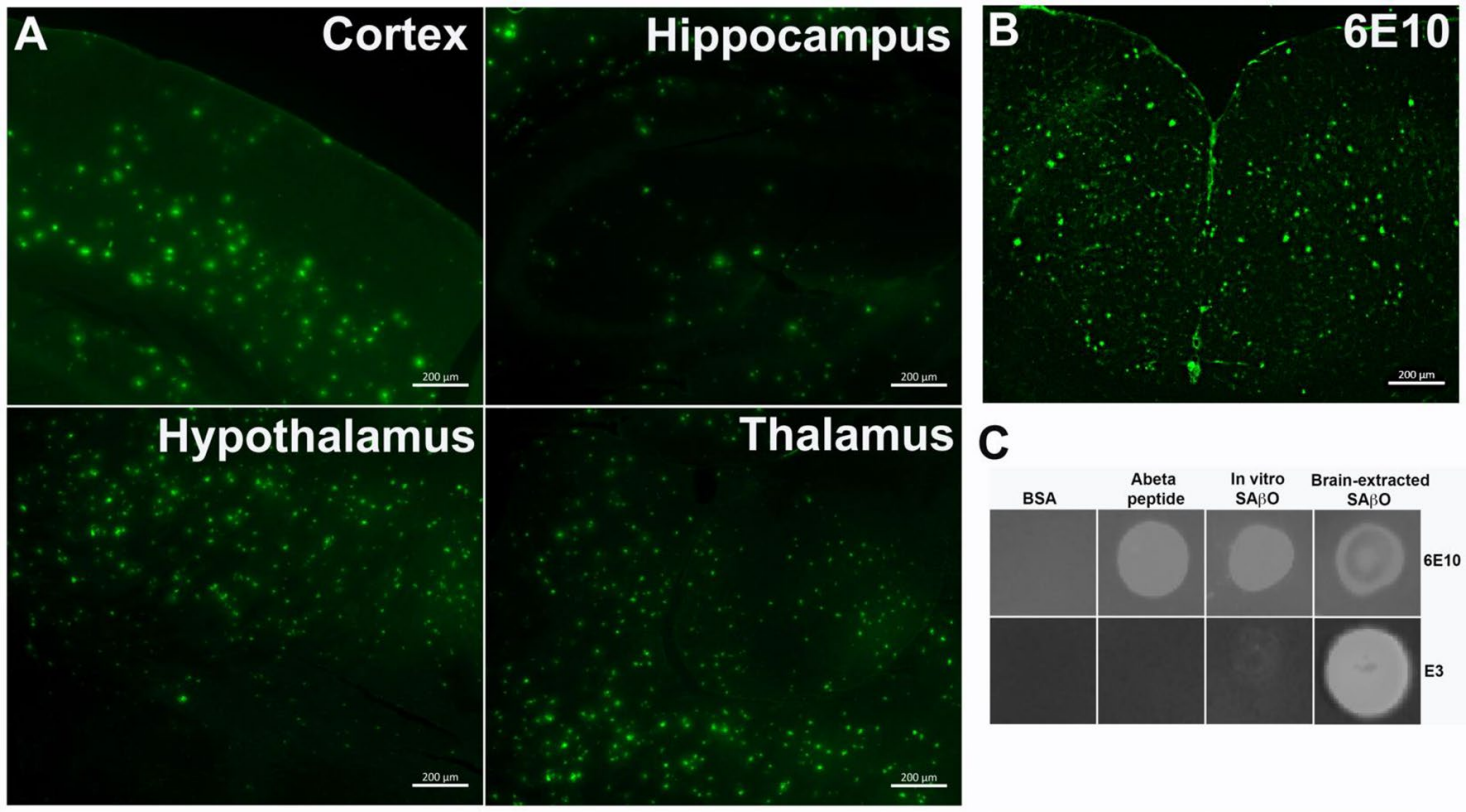
Specificity of E3 nanobody using ex-vivo brain specimens and dot-blots. (**A**) Representative ex-vivo imaging of different regions of the brains of 5XFAD mice (n = 3, 10-month-old) after staining with FAM-labeled E3 nanobody. (**B**) Ex-vivo staining of AF488-labeled 6E10 antibody on a 5XFAD brain slide. All ex-vivo staining of brain slides was incubated for 1 h at room temperature. (**C**) Dot-blot analysis to test the binding specificity of E3 nanobody against 6E10 antibody.

We used the FAM-labeled E3 nanobody to investigate the spatial distribution of SAβO in ex-vivo brain sections. The data in Fig. 5A demonstrate that the FAM-labeled E3 nanobody (1.8 μg/mL) stained several regions of the brains obtained from 5XFAD mice (n = 3, 10-month-old). The stained amyloid-β species are heterogeneous and have distinct size, shape, and morphology features. More dense populations are found within the hypothalamus and thalamus, followed by cortex (Table 1). Aside from the smaller size species (~ 2 μm), the large-sized amyloid-β species (~ 14 μm) detected with FAM-labeled E3 are similar to those detected by Alexa Fluor-488-labeled 6E10 antibody (Fig. 5B). The 6E10 staining data are consistent with a past report that described amyloid-β plaques in 5XFAD mice to be approximately 20 μm^49^. Using an in-house algorithm written in MatLab (Mathworks, Natick, MA), we analyzed the physical characteristics of amyloid species in the brain stained with FAM-labeled E3 nanobody. Overall, the density of amyloid-β species present in the thalamus and hypothalamus is higher than in the hippocampus or cortex (Table 1). It is also notable that the size of these SAβOs is nearly sevenfold smaller than that of amyloid-β plaques.

**Table 1:** Quantitative analysis of the amyloid-β species in the coronal brain sections of 5XFAD mice, depicted by the FAM-labeled E3 nanobody.

Brain regions	Number of animals	Number of observed brain slides	Total number of Abeta populations		Density (number/mm^2^)		Abeta area (μm^2^)		Average size in diameter of Abeta-associated FAM-E3^a^ (μm)		Eccentricity	
			Average	S.D	Average	S.D	Average	S.D	SAβO	Plaques	Average	S.D
Cortex	3	21	131.86	51.69	89.72	38.28	68.23	123.96	2	14	0.52	0.28
Hippocampus	3	6	63.00	62.61	38.87	34.74	41.01	87.95			0.57	0.27
Hypothalamus	3	6	296.50	7.78	170.90	3.10	47.69	77.34			0.63	0.23
Thalamus	3	6	207.75	78.49	122.44	46.68	43.92	78.02			0.53	0.29

^a^Determined using ImageJ.

Next, we developed a dot blot assay to test the specific recognition of E3 nanobody versus 6E10 antibody for different amyloid-β species, including free amyloid-β peptide, extracted SAβO from 5XFAD brains, or the in-vitro produced counterparts (Fig. 5C). For this study, the amyloid-β peptide was prepared fresh using human amyloid-β (1–42) peptide, while extracted SAβOs were obtained from the supernatant of homogenized 5XFAD mouse brains after ultracentrifugation. The in-vitro SAβO was generated as described in Fig. 2. All three samples of the amyloid isoforms were quantified by a BCA assay, enabling all isomers to be “spotted” at equal concentrations. The dot blot data (cropped and magnified for presentation) suggests that the E3 nanobody recognizes in-vitro SAβOs weakly. In contrast, it recognizes extracted SAβOs from 5XFAD brains strongly. The original dot blots are shown in Fig. S6 (Supplementary Information). This study also showed that the 6E10 antibody is less specific compared to the E3 nanobody as the antibody recognizes all three amyloid species.

### FAM-labeled E3 nanobody can cross the BBB and detect SAβOs and amyloid-β plaques in the brain after intravenous injection

To determine if the E3 nanobody could be used to image SAβO in-vivo, we injected the FAM-labeled E3 nanobody (1.8 mg/mL, 150 μL) into 5XFAD mice (n = 5) and WT (n = 3) mice. To ensure the fluorescent signal detected was not due to free dye diffusion to the brain or autofluorescence generated from the tissue or nanobody, two additional control cohorts of animals were included in the study. These controls included WT and 5XFAD mice that were intravenously injected with either free FAM dye (1 mg/mL, 150 μL) or unlabeled E3 nanobody (1.8 mg/mL, 150 μL) (n = 3, each), respectively.

**Figure 6:**
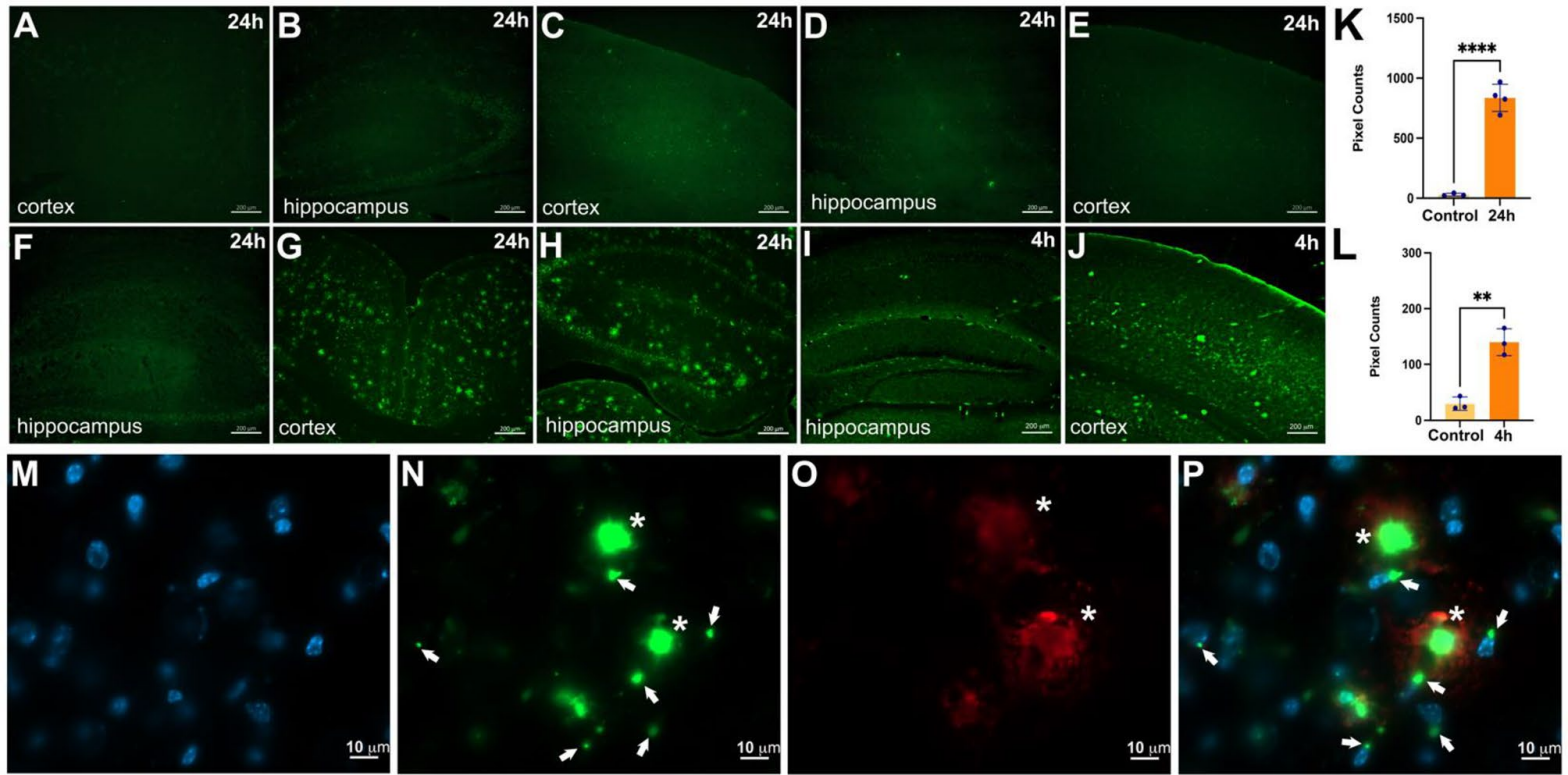
The E3 nanobody can cross the BBB and stain amyloid targets. Representative data of hippocampus and cortex per cohort. All treatments were performed by intravenous injection via the tail veins. 5XFAD mice (n = 3) were treated with E3 nanobody (**A**, **B**), resulting in no labeling. WT mice (n = 3) were treated with free FAM fluorescent dye (**C**, **D**), resulting in no labeling. WT mice (n = 3) were treated with FAM-labeled E3 nanobody (**E**, **F**), resulting in no labeling. 5XFAD mice (n = 4) were treated with FAM-labeled E3 nanobody 24 h before brain collection (**G**, **H**), resulting in significant numbers of bright green fluorescent profiles in cortex and hippocampus. 5XFAD mice (n = 3) were treated with FAM-labeled E3 Nb 4 h before brain collection (**I**, **J**), resulting in fewer bright green fluorescent profiles. The fluorescent pixels were quantified and compared between (**A**, **B**) (n = 3) versus (**G**, **H**) (n = 4) at ****p < 0.0001 (**K**). And between (**A**, **B**) (n = 3) versus (**I**, **J**) (n = 3) at *p  < 0.005 (**L**). Confocal imaging of ex-vivo 5XFAD hippocampus slides: DAPI staining of the nucleus (**M**). FAM-labeled E3 recognized SAβO (white arrows) and larger amyloid-β plaques (asterisks) (**N**). Amyloid-β plaques stained by Alexa 647-labeled 6E10 antibody (**O**). Merging of M, N and O (**P**) indicates E3-positive staining around the nuclei (white arrows).

We imaged many different areas of the brain, but chose to present the cortex and hippocampal regions for each cohort since they are relevant to AD. As shown in Fig. 6, no fluorescent signals were detected in 5XFAD mice injected with E3 nanobody alone (Fig. 6A,B) or in WT mice injected with either free FAM dye (Fig. 6C,D) or FAM-labeled E3 nanobody (Fig. 6E,F). In contrast, FAM-labeled E3 nanobody detected copious amyloid-β deposits in the brain of 5XFAD mice at 24 h (Fig. 6G,H) and 4 h (Fig. 6I,J) post-injection. Pixel quantification the signals demonstrated that the 24 h-post-injection group was significantly higher than that of control mice (Fig. 6K) (*p* < 0.0001). Although by a smaller amount than the 24 h-cohort, the accumulation of signal in the 4 h-post-injection cohort (n = 3) was significantly higher than control counterparts (*p* < 0.005) (Fig. 6L).

With the superior resolution provided by confocal microscopy, we also showed that SAβO and amyloid-β plaques recognized by the E3 nanobody not only differ in size, shape, and morphology, but they have distinct spatial distributions in the brain. As shown in Fig. 6M–P**,** SAβOs are associated with the neuronal nuclei (white arrows). This observation agrees with a previous report showing the association of oligomers with neuronal nuclei^50^. In contrast, the E3 nanobody-positive amyloid-β plaques (asterisks) reside in the cellular matrix. We also confirmed that E3 nanobody binds amyloid-β plaques isolated from 5XFAD brains in a dot blot study (Fig. S7). These results indicate that E3 nanobody, similar to other nanobodies, crosses the BBB, most likely due to its small size and relatively high isoelectric point (calculated to be 9.2 using Expasy^43,44,51,52^).

### Quantitative analysis

The in-vivo data obtained using FAM-E3 nanobody revealed the spatial distribution of SAβOs in 5XFAD brains. In contrast, no registered fluorescence signal was detected in WT brains. While SAβOs and amyloid-β plaques were distributed across many regions of the brain of 5XFAD mice, they appeared to be more concentrated in some brain regions than others. Thus, we were interested in quantifying these differences using our algorithms developed in MATLAB. The data are summarized in Table 1 and show that the presence and the density of amyloid-β species in the hypothalamus (170.90/mm^2^) was surprisingly high, followed by the thalamus (122.44/mm^2^), compared to other regions. On the other hand, the hippocampus had the least number of amyloid-β species with a recorded density of 38.87/mm^2^). On average, the diameter of SAβO-associated FAM-E3 was about 2 μm, with average measured eccentricities of approximately 0.5.

## Discussion

The accumulation of amyloid-β aggregates in the brain is a hallmark of AD. In human AD, pathological amyloid-β proteins are composed of different assemblies that included senile plaques, protofibrils, fibrils, and SAβOs. Increasing evidence suggests that SAβOs serve as toxic species responsible for the pathogenesis and spread of AD^50^. Prior to this work, a number of antibodies^50,53^ or antibody fragments^54^ have been used for the specific recognition of SAβOs. These reagents have helped examine the roles of SAβOs in AD brain, leading to the hypothesis that the disruption of particular synapses by SAβOs could be the culprit behind the memory loss in early AD^8^. According to the contemporary model of the amyloid-β cascade hypothesis (Fig. 1), SAβOs are considered stable and independent components, affecting AD pathology without conversion to fibrillar assemblies^4^. Thus, targeting SAβOs is beneficial due to their early role in AD pathogenesis and disease progression. In this work, we report the development, production, and characterization of a novel SAβO-specific nanobody. Aside from being thermally stable, which makes it amenable for PET labeling chemistry, a nanobody has the added advantage of being able to traverse biological barriers. With a mere size of ~ 15 kDa, 1/10th the size of conventional antibodies (150 kDa), nanobodies are smaller than any antibodies and antibody fragments described to date. For example, Fab (~ 50 kDa), F(ab’)_2_ (~ 110 kDa fragments), single chain (monovalent) Fv (scFv, ~ 25 kDa) with one antigen-binding site, bivalent scFv dimmers (50 kDa), and scFv-fusion proteins (sometimes called minibodies, ~ 80 kDa)^55^, all are larger than nanobodies. We anticipate that using nanobodies for targeting SAβOs could serve as a gambit for responding to the delivery solution for biologics. These molecules could be used either as therapeutics or diagnostic pharmaceuticals for AD applications.

Nanobody production is relatively simple, robust, and economical. The quality of the SAβO antigen used for immunization was confirmed by Coomassie staining and Western blot analysis (Fig. 2A,B). We prepared relatively fresh SAβOs for alpaca injections, thus the material had a low propensity to grow larger aggregates. The VHH library was constructed, and a single panning step was performed against the immobilized SAβOs. From the enriched SAβO nanobody-expressing phage, we identified 61 clones by screening for binding to SAβOs via ELISA.

We also developed a robust assay to screen nanobody FAM-labeled nanobody clones using consecutive 5XFAD brain slides. Data in Fig. 4 showed that no signals were detected in the WT brain sections. In stark contrast, all the clones stained oligomers in the hippocampus and cortex of a 5XFAD mouse, although the degree of staining varied between clones in the brain slices. Among the six best clones, D11, F9, H5 and E3 showed considerable signal with at least 100 pixel counts per cortex, as we quantified using ImageJ. From these results, we picked the E3 nanobody for full characterization. We repeated the ex-vivo staining with FAM-labeled E3 nanobody using brain slides from different 5XFAD mice (n = 3). The data showed that the FAM-E3 nanobody stained amyloid-β in the 5XFAD brains with a mixed morphology (Fig. 5A). It is apparent that two populations of amyloid-β isoforms dominate many parts of the brain, with the highest abundance found in the hypothalamus, followed by the thalamus, cortex, and then hippocampal regions (Table 1). The size, shape, and morphology of the smaller population are similar to what has been seen with anti-SAβO ACU193 and NU4 antibodies^56^. Meanwhile, the larger population observed in the 6E10 antibody-treated 5XFAD brain slides resembles amyloid-β plaques (Fig. 5B). To explain the mixed results of our in-vivo amyloid-β staining, we tested the specificities of E3 nanobody against amyloid-β isomers that included monomers, SAβOs, in-vitro generated SAβO, and SAβO, which were isolated from 5XFAD brain homogenates using ultracentrifugation. The results of this work confirmed the ex-vivo staining data showing that E3 nanobody is more specific than 6E10 antibody for SAβO in that it can recognize SAβOs extracted from the brain of 5XFAD mice but not the free amyloid-β peptide monomer (Fig. 5C). The data suggest that the nanobody recognizes the SAβOs not only based on the specific epitope but depending also on the secondary structure. A complete understanding of the molecular mechanism by which E3 nanobody preferentially recognizes oligomeric forms of amyloid- will require detailed structural analysis. However, an enticing possibility is suggested by AlphaFold models of E3. The complementary determining regions of E3 may make a large, relatively flat surface, capable of binding across multiple rungs of the beta-solenoid structure of amyloid oligomers (Fig. S8, Supplementary Information). Such a binding mechanism would lead to strong preference for oligomers over monomers.

Importantly, the E3 nanobody could localize in the brain after intravenous injection. The in-vivo application of FAM-labeled nanobody (via intravenous injection) indicated that it could cross the BBB and stain amyloid-β in the brain of 5XFAD mice (Fig. 6). The FAM-labeled E3 nanobody is distributed significantly in the brain of 5XFAD mice, 4 h post-intravenous injection (Fig. 6I,J). These data point to an advantage of nanobodies compared to larger antibodies. It has been reported that an uptake time of 4 h post-intravenous injection of anti-SAβO antibody labeled with ^64^Cu PET radioisotope in 5XFAD mice resulted in poor signal distribution and was not robust for signal detection^56^. In our work, we observed increased signal in the 24 h-post-injection cohort (Fig. 6G,H). Based on the pharmacokinetics of the probe, it appears that the probe enters the brain of injected mice, binds SAβO targets and accumulate, leading to increased signal. The confocal imaging data showed the distinct locations between SAβOs (white arrows) and amyloid-β plaques (asterisks) (Fig. 6M–P). The former is associated with neurons, while the latter resides in the extracellular matrix. This regiospecific distribution may explain the distinct role of how they contribute to AD pathology.

In conclusion, we report a robust, large-scale, and reproducible generation of a novel SAβO nanobody. Extensive in-vitro and in-vivo characterization demonstrated that the nanobody recognizes SAβOs and amyloid-β plaques in a preclinical mouse model of AD. Since FAM-labeled E3 can penetrate the BBB and effectively stain amyloid-β in the brain, work is currently being undertaken to utilize this nanobody for diagnostic applications and potential therapies. Because SAβOs are involved in the early stage of AD pathology^21^, we anticipate that this nanobody can be utilized to develop a novel and powerful platform for precision in-vivo molecular fingerprinting of SAβOs for AD early detection.

## Methods

### Materials

All commercially-available reagents and solvents were used as received without further purification. FAM-NHS was purchased from Lumiprobe Corp (Hunt Valley, Maryland, USA), sealed with parafilm and stored at − 20 °C. The purity and stability of the dye were monitored every 6 months using liquid chromatography mass spectrometry (LC-MS). Human amyloid-β peptide (1–42) was obtained from Anaspec (Fremont, CA, Cat. AS-20276). Ultrapure water (18.2 MΩ) was obtained from a Millipore Direct Q-5 water purification system. A Hitachi HPLC system (Lachrom Elite) equipped with a diode array detector was used for purification, and employed a Cytiva HiTrap Desalting column with a mobile phase of 25 mM sodium phosphate, 150 mM sodium chloride, pH7.4. Three fluorescence microscopes were used in our studies, including a manual Zeiss Axio Observer, an automated Zeiss Axio Observer, and a Zeiss LSM 710 confocal laser scanning microscope. MALDI-TOF analysis was performed on a Bruker Rapiflex with sinapinic acid matrix.

### Animals

All animal experiments performed complied with institutional guidelines and were conducted according to the protocol approved by the Vanderbilt Institutional Animal Care and Use Committee. Furthermore, all work performed in this study complies with the Animal Research Reporting In Vivo Experiments (ARRIVE) guidelines. The animals were maintained at Vanderbilt University under standard conditions, in a 12 h-light/dark cycle, and with free access to food and water, as we described in the past^57^. The 5XFAD mice overexpress both mutant human APP and PS1 genes and exhibit high APP levels correlating with high burden and accelerated accumulation of amyloid-β^57^. The mice were genotyped by a standard polymerase chain reaction (PCR) using DNA isolated from tail tips and the following primers provided by Jackson Laboratory (Bar Harbor, Maine): Mutant reverse, 5’-CGG GCC TCT TCG CTA TTA C -3’; Common, 5’-ACC CCC ATG TCA GAG TTC CT -3’; Wild Type reverse, 5’-TAT ACA ACC TTG GGG GAT GG-3’. Amplified PCR products were then analyzed by size fractionation using agarose (1.5%) gel electrophoresis; band sizes for wild type = 216 bp, 5XFAD heterozygous = 129 bp and 216 bp, and 5XFAD homozygous = 129 bp. The 5XFAD mice were maintained as heterozygotes and all mice used in these experiments were approximately 6- to 11-month-old.

Alpaca immunizations and bleeds were performed at Turkey Creek Biotechnology, LLC (Waverly, TN) in accordance with their Institutional Animal Care and Use Committee protocol 18-02.

### Intravenous injection via the tail vein

An animal was anesthetized with isoflurane (2%) under a flow of oxygen for 10 min. The animal is allowed to remain in deep anesthesia for approximately 10–20 s before commencing the procedure. The completely unconscious mouse will be checked with a stimulus, i.e., toe pinch. Then, the tail was cleaned with a non-alcoholic gauze pad, followed by warming the lateral veins using a flexible strip heater. Free or labeled E3 nanobody was injected with a catheter fitted with a 29-G needle tip. The volume is approximately 0.1 mL. After the procedure, the injection site was cleaned with an alcohol pad, and the animal was removed from anesthesia and returned to a warm cage for recovery.

### Generation of SAβO as an antigen

This work was performed using a reported procedure with some modifications^47^. Human amyloid-β peptide (1–42) (5.0 mg, Anaspec, Fremont, CA, Cat. AS-20276) was dissolved in DMSO to a final concentration of 5 mM. An equivalent volume of DMSO served as a negative control. The materials were sonicated at room temperature for 10 min in Eppendorf tubes. A small portion of the solution (20 μL) was set aside and stored at − 20 °C for future monomer analysis. The remaining solution was diluted further to 100 μM with cold PBS (1x) containing 0.05% SDS followed by a brief vortex (30 s). At the same time, the DMSO negative control was diluted with an equivalent of volume of cold PBS (1x) containing 0.05% SDS. The resulting stock solution of 100 μM peptide solution and control DMSO were then incubated overnight at 4 °C. Approximately 24 h later, the stock solution was further diluted to 25 μM with PBS (1x), and an equivalent volume of PBS (1x) was again added to the DMSO negative control. The samples were incubated for two weeks at 4 °C to enable amyloid-β oligomerization.

Following incubation, the amyloid-β concentration was found to be ~ 150 μg/mL as determined by the BCA protein assay (Thermo Fisher Scientific, Waltham, MA, Cat. 23225). The amyloid-β was then concentrated using Amicon Ultra-4 Centrifugal Filter Units with a cutoff of 3 kDa NMWL (Millipore Sigma, Burlington, MA, Cat. UFC800324) to reach a final concentration of 500 μg/mL. The purity of the SAβO was confirmed by SDS-PAGE using 12% precast polyacrylamide gels (BioRad, Hercules, CA, Cat. 4561044) followed by Coomassie staining (Fig. 2A**)**. The data showed that with this method, we could generate a mixture of low and high molecular weight oligomers. Freshly prepared samples were made prior to each animal immunization, and the quality of each preparation was confirmed by SDS-PAGE and Coomassie staining of the gels.

### Coomassie staining

Soluble amyloid-β oligomer preparations were solubilized in Laemmli Sample buffer (1X final concentration), boiled for 5 min at 96 °C, and analyzed by SDS-PAGE. The prepared samples (20 μL) were loaded into a 12-well 4–20% gradient gel (BioRad Laboratories, Hercules CA, Cat No. 4561095) along with BioRad Precision Plus Protein Kaleidoscope Prestained Protein Standards (BioRad Laboratories, Hercules CA, Cat No. 1610375) and electrophoresed at 170 V for 50 min. The gel was washed three times for 5 min each with deionized water, stained with BioSafe Coomassie G-250 (BioRad Laboratories, Hercules CA, Cat No. 1610786) with gentle shaking for 1 h, destained overnight, and imaged on a LiCOR Odyssey M (LiCOR Biosciences, Lincoln NE). Images were collected and extracted using Empiria software (LiCOR Biosciences, Lincoln NE).

### Western analysis

Samples were prepared for immunoblotting by adding Laemmli Sample buffer (1X final concentration) and 20 μL of each sample was loaded onto a 12-well 4–20% gradient gel (BioRad Laboratories, Hercules CA, Cat No. 4561095) along with BioRad Precision Plus Protein Kaleidoscope Prestained Protein Standards (BioRad Laboratories, Hercules CA, Cat No. 1610375). Following electrophoresis at 170 V for 50 min, the gel proteins were transferred onto a 0.2 μm supported nitrocellulose membrane (BioRad Laboratories, Hercules CA, Cat No. 1620097) using the TransBlot Turbo System (BioRad Laboratories, Hercules CA, Cat. No. 1704150) and TransBlot Turbo Buffer (BioRad Laboratories, Hercules CA, Cat No. 10026938) at 2.5A, 25 V, for 10 min. The blots were rinsed in deionized water for 5 min, then placed between two pieces of Whatman filter paper (Cytiva, Malborough MA, Cat No. 10427818) to dry for 15 min at 37 °C. Dried blots were rehydrated in TTBS for 5 min and blocked for 1 h in LiCOR Odyssey Intercept Buffer TBS (LiCOR Biosciences, Lincoln NE, Cat No. 27-60001) at room temperature. Blots were heat-sealed in ULine Poly Tubing bags containing 5 mL of 1:1000 (v/v) 6E10 antibody (BioLegend, San Diego CA, Cat No. 803004) in ULine Poly Tubing and incubated overnight at 4 °C. Blots were then washed 4 times with large volumes of TTBS, and incubated with 1:5000 (v/v) LiCOR Donkey anti-Mouse IRDye800CW (LiCOR Biosciences, Lincoln NE, 926-32212) for 1 h at room temperature. Blots were washed 4 times with TTBS and imaged on a LiCOR Odyssey M LiCOR Odyssey M (LiCOR Biosciences, Lincoln NE) at 800 nm and 700 nm channels, and images were collected and processed using LiCOR Empiria Software (LiCOR Biosciences, Lincoln NE).

### Alpaca immunization

The SAβO preparation (300 μg) was emulsified in Gerbu adjuvant for the initial injections (day 0), followed by boosters at days 14, 28, 42, 56, 70 and 84 with 150 μg SAβO in Gerbu adjuvant. Blood was drawn for analysis on day 63 (first bleed) and day 91 (second bleed). The plasma was monitored by dot-blot analysis to test for the presence of antibodies recognizing SAβO. Peripheral blood mononuclear cells (PBMCs) were isolated by centrifugation from 17.5 mL of alpaca blood using SepMate centrifugal devices, following the manufacturer’s protocol (Stemcell Technologies, Vancouver, CA).

### Phage library construction

A cDNA library was made by reverse transcription with oligo dT primers and Superscript IV (ThermoScientific). A nested PCR strategy was used to amplify VHH coding regions of fragments, as described^48,58^. The resulting PCR fragments were ligated into pBBR3, a modified pADL22 vector (Antibody Design Labs). All clones contain in-frame, C-terminal, HA and hexahistadine tags. The library was electroporated into high-efficiency TG1 cells (Lucent), and phage were produced using CM13 helper phage. Phage were harvested from the supernatant by PEG precipitation and suspended in PBS.

### Panning for SAβO nanobodies and isolation of individual clones

A single round of panning against SAβO immobilized on a Nunc MaxiSorp (Nalge Nunc Intl., Rochester, NY) plates was performed. Three wells of a 96-well MaxiSorp plate were coated overnight with 10 μg of SAβO in PBS (100 μL); three PBS-coated wells served as controls. After removing unbound antigen, the wells were blocked with 2% nonfat milk in PBS for 2 h, washed, and incubated with 2 × 10^12^ phage particles in blocking buffer for 1 h. After extensive alternating washes with PBS and PBS + 0.5% Tween 20, phage were eluted with 100 µL of 100 mM glycine pH2.2 and then immediately neutralized with 100 μL of 1 M Tris pH8. Recovered phage were used to infect TG1 *E. coli*, and single clones were picked and inoculated in deep-well 96 well blocks containing 1 mL Terrific Broth and 100 μg/mL ampicillin. Plates were grown at 37 °C for 5 h, followed by 28 °C overnight. Then, 1 mM IPTG was added, and the growth was continued for 3 h. Bacteria were pelleted and lysed by two freeze–thaw cycles with a total of 400 µL of PBS. After centrifugation, the periplasmic extracts were analyzed by ELISA for the presence of nanobody-recognizing SAβO.

### Detection of positive SAβO nanobody clones

A MaxiSorp plate was coated overnight at 4 °C with 1 µg/well of SAβO in PBS (100 μL), washed, blocked with LI-COR Intercept buffer (PBS), and then incubated with 50 µL of periplasmic extracts lysate from above and 50 µL of PBS for 2 h at room temperature or overnight at 4 °C. Wells were washed with TTBS and incubated with a HRP-conjugated anti-HA tag (Roche, Basel, CH) antibody for 1 h at room temperature. After washing, the plate was developed with TMB ultra (Sigma, St. Louis, MO) to identify positive clones.

In an alternative procedure, a 96-well Costar plate (black with clear bottom) was coated overnight at 4 °C with 0.5 μg/well of SAβO (100 μL), washed with TTBS, blocked with LI-COR Intercept buffer (TBS), and then incubated with 50 μL of periplasmic extracts from above and 50 μL of PBS for 2 h at room temperature or overnight at 4 °C. The wells were washed with TTBS and incubated with fluorophore 800-conjugated anti-HA antibody for 1 h at room temperature. After washing with TTBS, the plate was imaged using the LI-COR Odyssey Imager to identify positive clones.

### Clone identification

A total of 4 96-well plates of clones were picked from two different nanobody libraries. All clones showing signal above background were sequenced. Six clones were both identified multiple times and found to be positive in multiple assays. These six clones were expressed and purified from 3 L bacterial cultures. A portion of each purified nanobody was biotinylated with 20-fold molar excess of NHS-biotin (Pierce) and 8 point, fivefold, binding titrations were done for each clone using streptavidin-HRP or anti-HA-HRP (Thermo Fisher).

### Nanobody purification

For large scale purifications of E3, the plasmid was isolated and used to transform T7 shuffle bacteria. An overnight culture was used to inoculate fresh media which was grown at 37 °C until a OD600 of ~ 1.2 was reached. At this point the temperature was lowered to 18 °C, 1 mM IPTG was added, and protein expression was continued overnight. Cells were harvested by centrifugation and lysed with an Emulsiflex. Nanobodies were purified using Talon affinity resin (Takara Bio, Ann Arbor, MI). Aliquots of E3 were chemically biotinylated using NHS-Biotin (Thermo Fisher, Waltham, MA).

### Labeling E3 nanobody with FAM-NHS dye

The E3 nanobody (1.8 mg/mL, 57 nmol) in PBS (1x, 50 μL) was treated with Na_2_CO_3_ (1 M, 6 μL, pH8) prior to the addition of FAM-NHS dye (20 eq., 1140 nmol, 0.54 mg) in argon-purged anhydrous DMSO (12 μL). The reaction vial was rotated overnight at 4ºC. Excess of activated dye was neutralized with Tris-HCl buffer (10 μL, pH7), and the labeled product was separated from the excess dye using a 7 k MWCO Zepa spin column and reconstituted in PBS (1x, pH7). The resulting FAM-E3 conjugate was characterized by 2D-HPLC using a Cytiva HiTrap desalting column (5 mL, cat. 29048684) under the isocratic conditions of 25 mM sodium phosphate, 150 mM sodium chloride, pH7.4, and a flow rate of 0.5 mL/min.

### Immunohistochemistry staining

Brains embedded in OCT were cut into coronal Sects. (10 µm) using a Tissue-Tek (Sakura Finetek USA, Torrance, CA) cryostat and mounted onto charged glass slides. Prior to staining, slides were washed with PBS (10 min) and then treated with blocking buffer (5% normal goat serum, 0.2% Triton X-100, 0.5% bovine albumin in PBS) for 1 h at room temperature. The treated sections were then incubated overnight at 4 °C or 1 h at room temperature with the E3-labeled nanobody diluted 1:100 in PBS. The sections were then washed with PBS twice for 10 min and once for 30 min, and cover-slipped with Vectashield plus antifade mounting medium containing DAPI (Vector Laboratories, Burlingame, CA, Cat. H2000-2) before observation using fluorescence microscopy.

For slides that required the use of secondary antibodies, the process was performed the same way as above except that after incubation with nanobody, the sections were subsequently washed in PBS 3 times for 10 min each and then incubated for 30 min at room temperature with a secondary antibody diluted in PBS containing 3% NGS (normal goat serum), 0.2% Triton X-100 and 0.5% bovine albumin. The sections were then washed with PBS twice for 10 min and once again for 30 min before being cover-slipped with Vectashield plus and examined by fluorescence microscopy.

### Isolation of amyloid-β fibrils/plaques from 5XFAD mouse brains

This procedure was modified based on the Abcam “Extraction of amyloid-β plaques from mouse brain” protocol (https://www.abcam.com/protocols/extraction-of-amyloid-beta-from-mouse-brain). The brain of a 5XFAD mouse (n = 2, 6-month-old) was homogenized in PBS (5 mL), followed by the addition of formic acid (10 mL). The mixture was sonicated for 1 min on ice and centrifuged at 135,000 g for 1 h at 4ºC. The collected supernatant was neutralized with a neutralizing buffer composed of 1 M Tris base, 0.5 M Na_2_HPO_4_, and 0.05% NaN_3_. The solution was aliquoted, and flash frozen on dry ice before storage at − 80 °C. Aliquots were thawed prior to loading onto nitrocellulose membranes for dot-blot analysis.

For extraction of SAβO, the brains of a 5XFAD mice (n = 3, 6-month-old) were homogenized in a solution of 0.2% diethylamine in 50 mM sodium chloride. Then, the suspension was centrifuged at 100,000 × g for 1 h at 4 °C. The supernatant containing SAβO was neutralized with 0.5 M Tris HCl, pH 6.8. Neutralized samples were frozen and flash-frozen on dry ice and stored at − 80 °C before use.

### Dot-blot assays

For testing the plasma, one microliter of 0.1, 0.01, 0.001, and 0.0001 mg/mL stock solutions of SAβO in SDS sample buffer or PBS were spotted on Amersham Protran Supported 0.2 mm Nitrocellulose (GE Healthcare Life Sciences). The membranes were allowed to dry, wetted in Tris-buffered saline (TBS), blocked in Intercept Blocking Buffer (LI-COR), and then incubated overnight with alpaca plasma diluted 1:200 in Intercept Blocking Buffer. After washing with TBS/0.1% Tween, the membranes were incubated with an 800 fluorophore-labeled goat anti-alpaca secondary antibody, washed with TBS/0.1% Tween, and then imaged on the Odyssey Infrared Imaging system (LI-COR).

For testing the specificity of the E3 nanobody, samples of bovine serum albumin (BSA), amyloid-β monomer, *in-vitro-generated* SAβO, and mouse brain SAβO extract were spotted onto a nitrocellulose membrane and allowed to dry at room temperature. The membrane was then blocked for 30 min using Intercept (TBS) blocking buffer (LI-COR Bioscience, Lincoln, Nebraska) before three 5-min washes with TTBS. Next, the blot was incubated for 30 min in a solution containing E3 nanobody (10 mg/mL) in TTBS before three 5-min washes with TTBS. The membrane was then incubated with goat anti-VHH antibody (12.5 mg/mL in TTBS; Jackson ImmunoResearch, West Grove, PA) for 30 min and washed with TTBS three times for 5 min each again. Finally, the blot was incubated with donkey anti-goat AF488 (2 mg/mL in TTBS; Abcam, Cambridge, UK) and washed three more times for 5 min each with TTBS before imaging. All incubations and washes were performed at room temperature with gentle shaking. A separate control blot to test for non-specific binding was prepared likewise as above, except that no E3 nanobody was used as a primary antibody against the samples. Imaging was performed on a ChemiDoc MP Imaging System (Bio-Rad, Hercules, CA).

### Quantitative analysis program written on MATLAB

Semi-automated quantification of amyloid-β burden was performed for each slide using a custom image analysis script written in MATLAB (Mathworks, Natick, MA). First, an image was loaded into the program. Second, adaptive threshold was used to decrease the impact of background noise. Third, a tissue mask was created based on that adaptive threshold and then cleaned up using a morphological opening. Finally, regions-of-interest (ROIs) were manually drawn, enabling us to avoid histological artifacts. From the ROI plaque masks, plaque morphologic parameters can be calculated using the function "regionprops". Plaque density was reported in plaques per mm^2^ and was defined as the number of plaques divided by the ROI area.

### Confocal imaging

The brain sections obtained from 5XFAD mice (n = 3) were incubated with a mixture of FAM-labeled E3 (1.8 mg/mL) or mouse 6E10 (BioLegend, San Diego, CA, USA) for 1 h at room temperature with gentle rocking. Then, the slides were washed with PBS three times before being incubated with goat anti-mouse IgG cross-adsorbed secondary antibody, Alexa Fluor 647 (Invitrogen, Waltham, MA, USA). The slides were then counter-stained with DAPI incorporated in Vectashield (Vector Laboratories, Newark, CA, USA). The slides were sealed with a coverslip before microscopy. The images were obtained with 20 × and 63 × objectives using a Zeiss LSM 710 confocal laser scanning microscope. Then, the images were reconstructed with Zeiss Zen Blue software.

### Protein structure analysis

AlphaFold^59^ as implemented through the AlphaFold Colab was used to calculate 20 models of E3 using 4 different starting seeds. The predicted value of the local distance difference test (pLDTT), a per residue confidence value output by AlphaFold, was used for the coloring scheme in panel C in supplemental Fig. S8, as described in^60^.

### Statistical analysis

We used t-tests to compare the differences between two groups. The data were expressed as mean with SD of the difference. The statistical significance of the differences between sample means was determined via unpaired t-tests, the results of which were considered significant when *P* < 0.05 as reported with GraphPad Prism software.

### Supplementary Information

Supplementary Information.

## Data Availability

The complete amino acid sequence of the E3 nanobody is available in GenBank, with the accession number BankIt2800678 SABO_E3 PP401722. The data will be available after March 13th, 2024.
