# Supplementary material for: Targeting soluble amyloid-beta oligomers with a novel nanobody
Source: Sci Rep. 2024 Jul 12;14:16086. doi: 10.1038/s41598-024-66970-6 (PMC11239946; doi:10.1038/s41598-024-66970-6)

**TARGETING SOLUBLE AMYLOID-BETA** **OLIGOMERS WITH A NOVEL NANOBODY**

Justin R. Haynes ^1,2^, Clayton A. Whitmore ^1,2^ William J. Behof ^1,2^, Charlotte A. Landman^1,2^, Henry H. Ong^3^, Andrew P. Feld^6^, Isabelle C. Suero^6^, Celeste B. Greer^4^, John C. Gore^1,2,4,7,8,9^, Printha Wijesinghe^5^, Joanne A. Matsubara^5^, Brian E. Wadzinski* ^6,8^, Benjamin W. Spiller* ^6,11^, and Wellington Pham* ^1,2,4,7,8,9,10,12^

^1^ Vanderbilt University Institute of Imaging Science, Vanderbilt University Medical Center, Nashville, TN 37232, USA

^2^ Department of Radiology and Radiological Sciences, Vanderbilt University Medical Center, Nashville, TN 37232, USA

^3^ Department of Biomedical Informatics, Vanderbilt University Medical Center, Nashville, TN 37232, USA

^4^ Vanderbilt Brain Institute, Vanderbilt University, Nashville, TN 37232, USA

^5^ Department of Ophthalmology and Visual Sciences, University of British Columbia, Vancouver, British Columbia, V5Z3N9, Canada

^6^ Department of Pharmacology, Vanderbilt University, Nashville, TN, 37232, USA

^7^ Department of Biomedical Engineering, Vanderbilt University, Nashville, TN 37235,

USA

^8^ Vanderbilt Ingram Cancer Center, Nashville, TN 37232, USA

^9^ Vanderbilt Institute of Chemical Biology, Vanderbilt University, Nashville, TN 37232,

USA

^10^ Vanderbilt Institute of Nanoscale Science and Engineering, Vanderbilt University, Nashville, TN 37235, USA

^11^ Vanderbilt Center for Structural Biology, Vanderbilt University, Nashville, TN 37235, USA

^12^ Vanderbilt Memory and Alzheimer’s Center, Vanderbilt University Medical Center, Nashville, TN 37212, USA

*Correspondence:

1. [wellington.pham@vumc.org](mailto:wellington.pham@vumc.org)

2. [brian.wadzinski@vanderbilt.edu](mailto:brian.wadzinski@Vanderbilt.Edu)

3. benjamin.spiller@vanderbilt.edu

**Figure S1**. Original Coomassie staining data (20 μL load). Lanes: **1**, ladder; **2**, SAβO batch 1 (loaded at 50 μg/mL); **3**, SAβO batch 2; **4**, SAβO batch 3 (sample was stored at -20°C for multiple years); **5**, SAβO batch 4 (loaded at 0.45 μg/μL); **6**, amyloid-β peptide (25 μM, loaded at 1/1000th); **7**, another batch of amyloid-β peptide (thawed many times, loaded at 0.1 μg/μL); **8**, SAβO batch 5; **9**, SAβO batch 6; **10**, SAβO batch 7 (injected into alpaca).

Lanes 1, 2, and 3 in **Fig. 2A** were cropped from lanes 3,4 and 6 of this original data, respectively.


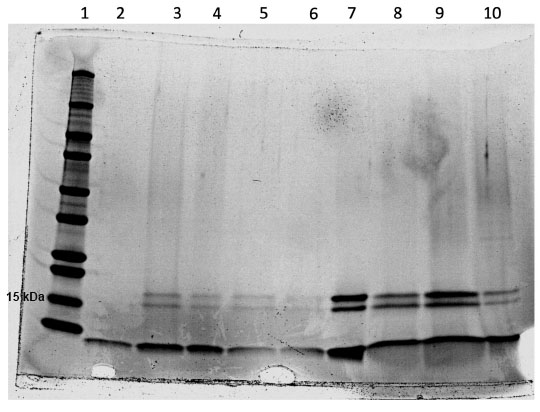


**Figure S2.** Original Western analysis to confirm the production of low molecular weight SAβO and some higher molecular species (15 μL load). Lanes: **1**, ladder; **2**, Formic acid extracted amyloid-β plaques/fibrils from 5XFAD brain lysate; **3**, SAβO batch 1; **4**, SAβO batch 2; **5**, SAβO batch 3; **6**, SAβO batch 4; **7**, amyloid-β peptide (25 μM) ; **8**, Another batch of amyloid-β peptide (thawed many times); **9**, SAβO batch 5; **10**, SAβO batch 6; **11**, SAβO batch 7 (injected into alpaca); **12**, we don't have a record of this lane.

Lanes 1, 2, and 3 of the Western blot in **Fig. 2B** were cropped from lanes 4, 5, and 7 of this original data, respectively.


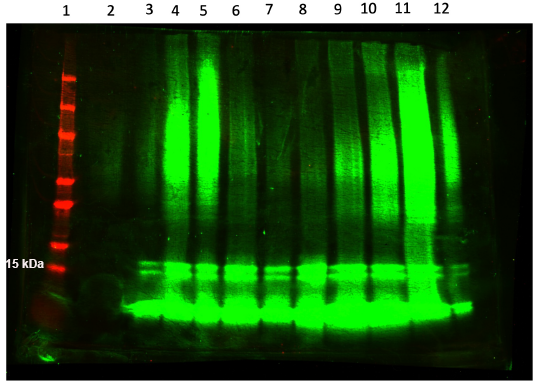


**Figure S3**. (A) Representative ELISA results from nanobody supernatants; (B) A total of 4 96 well plates worth of clones were picked from two different nanobody libraries. Six clones were both identified multiple times and positive in multiple assays. These six clones were expressed and purified from 1L bacterial cultures. A portion of each purified nanobody was biotinylated with a 20-fold molar excess of NHS-Biotin (Pierce), and 8-point, 5-fold, binding titrations were done for each clone using Streptavidin-HRP or anti-HA-HRP (Thermo Fisher).


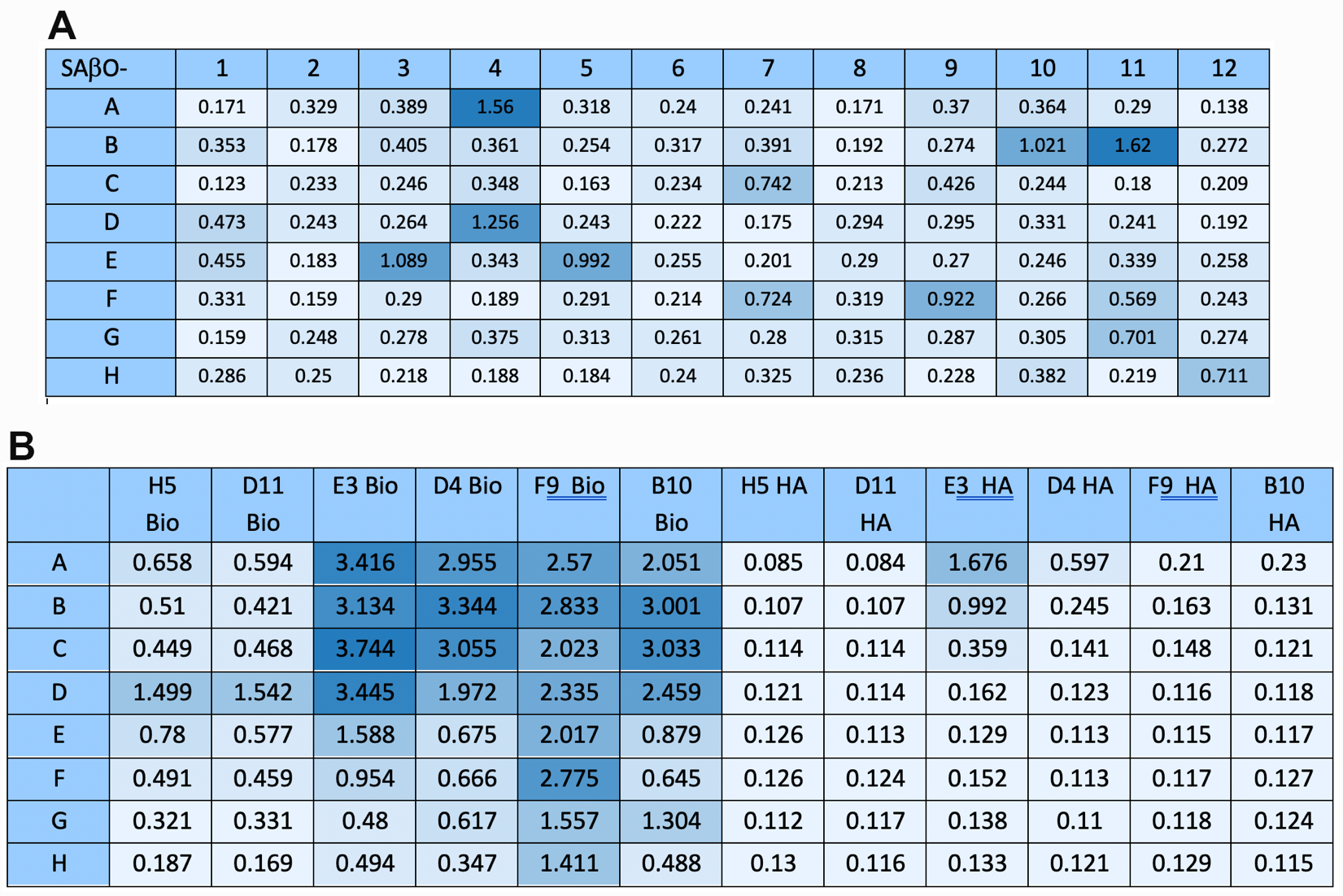


**Figure S4**. Western blot data showing successful FAM dye labeling of E3 nanobody, uncropped image. Lanes are, from left to right: Precision Plus Protein Kaleidoscope Standard Ladder (Bio-Rad), E3 nanobody, FAM-labeled E3 nanobody. This blot was probed with anti-HA 800 to detect nanobody signal and imaged for AF647 and DyLight 800 on a Bio-Rad ChemiDoc MP imaging system. Visible ladder bands (solid red bands) represent, from top to bottom: 250, 150, 100, 50, 20, and 15 kD, respectively.

**
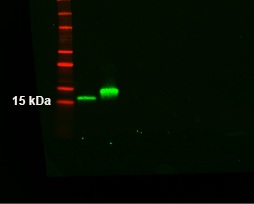
**

**Figure S5. Enlarged images of Fig. 4**

**B10: WT**

**
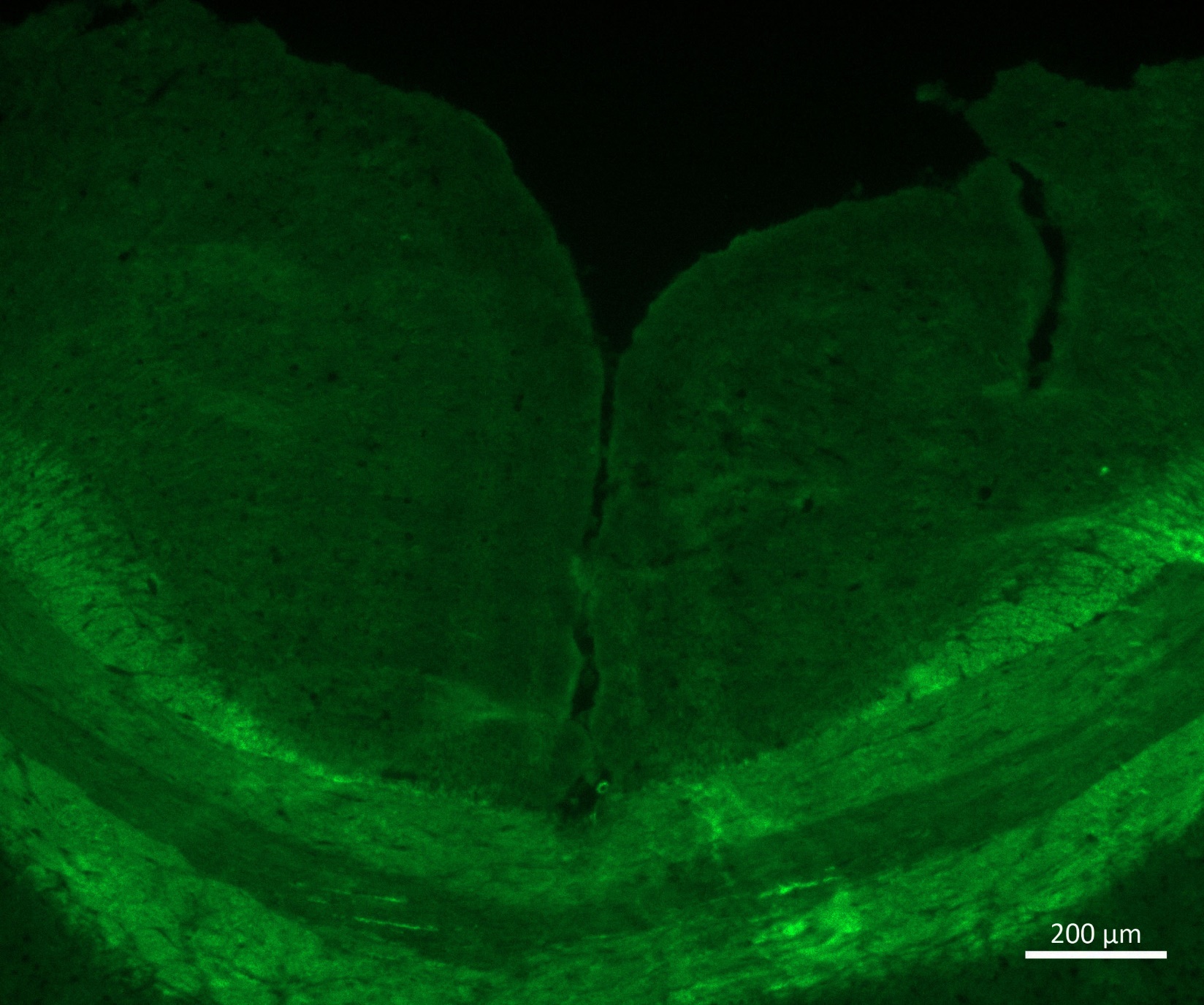
**

**B10: 5XFAD**

**
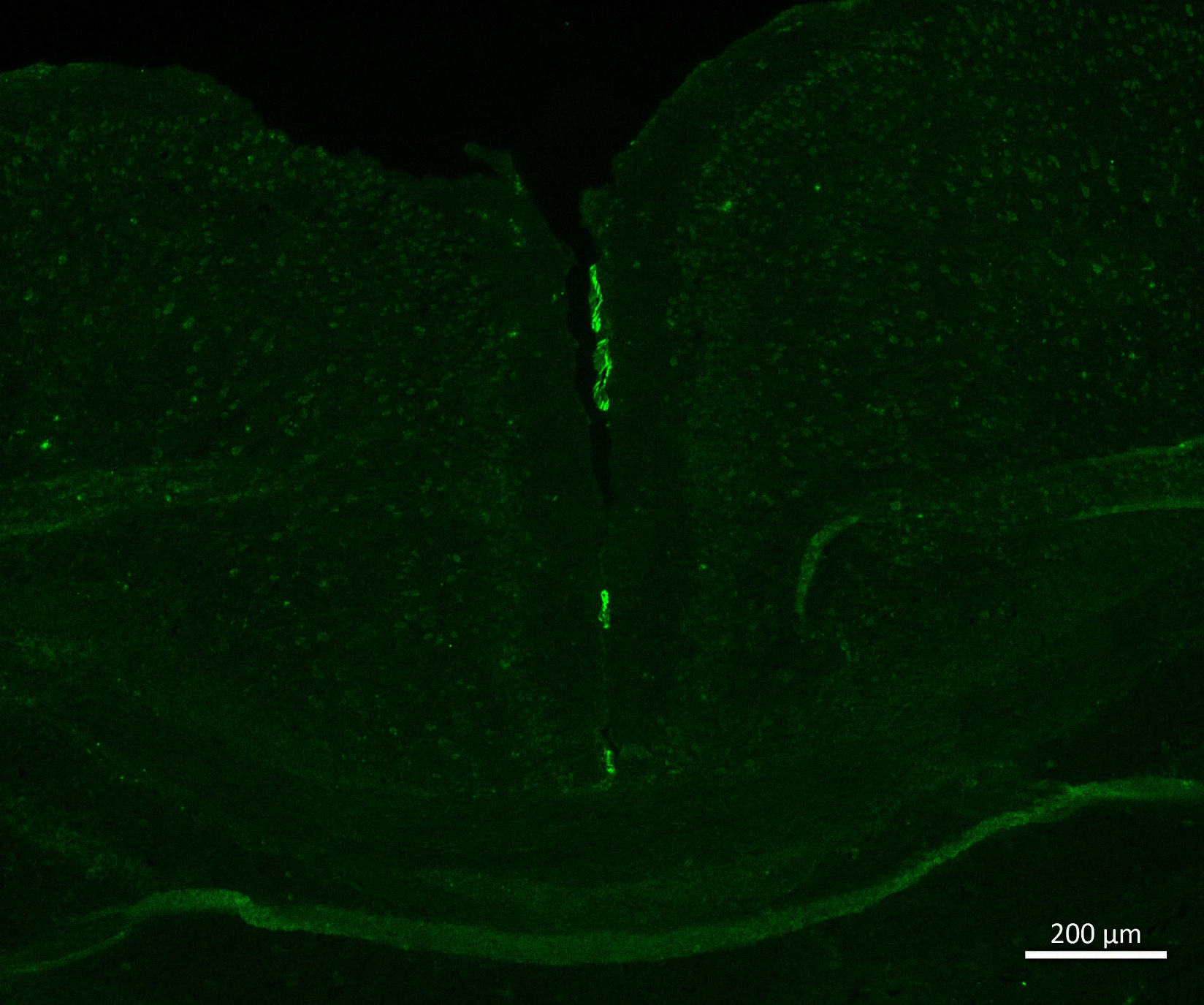
**

**D4: WT**

**
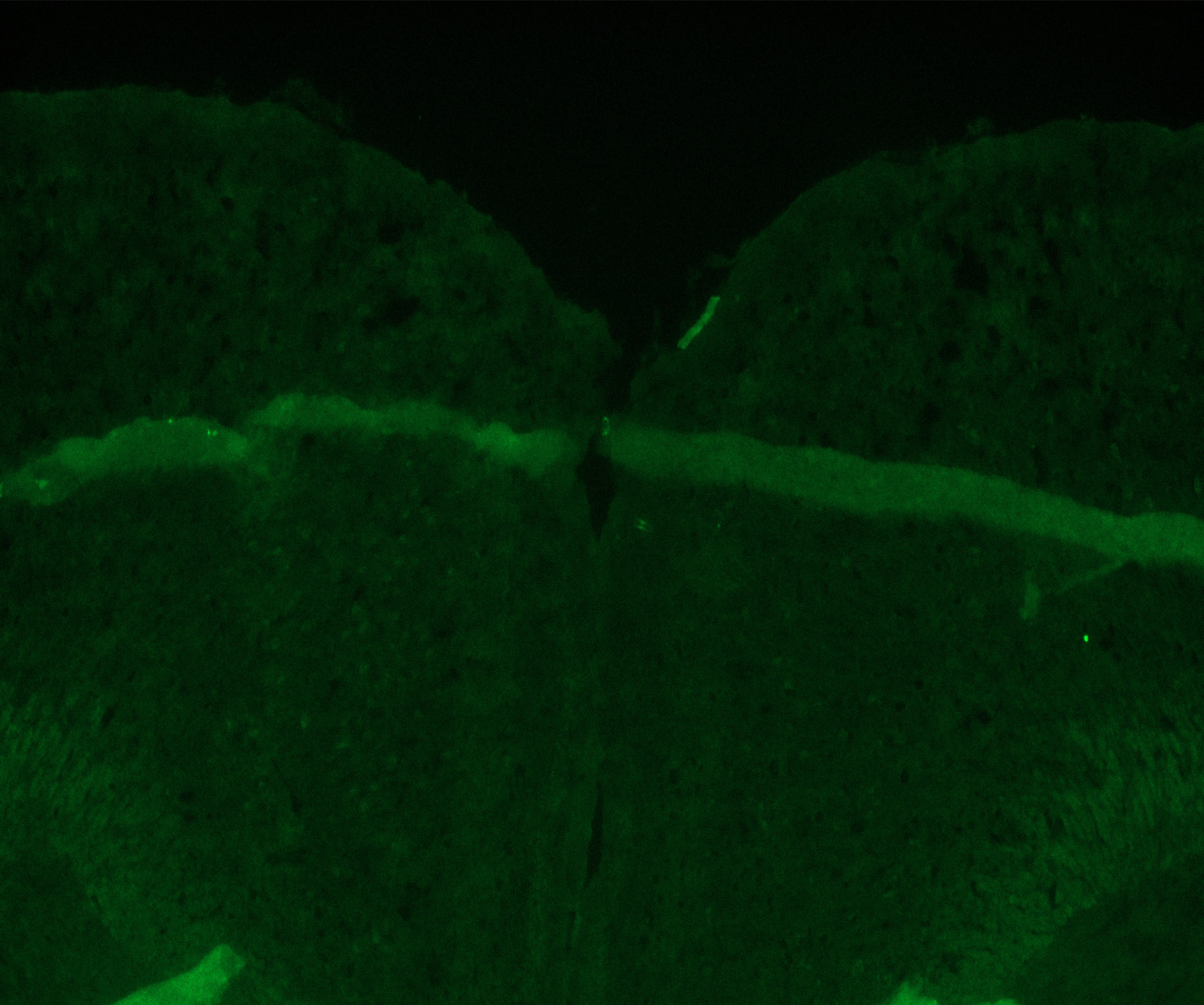
**

**D4: 5XFAD**

**
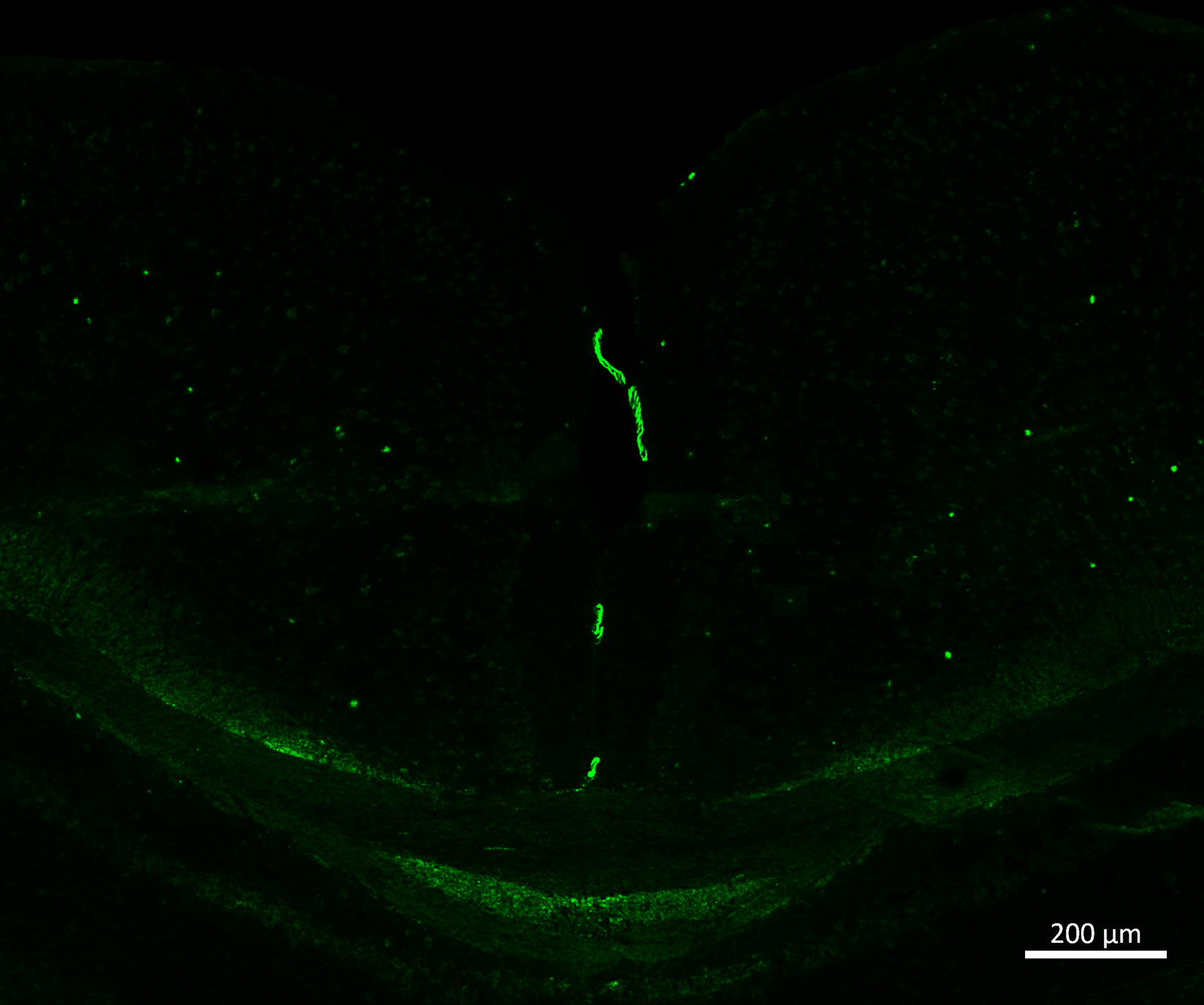
**

**D11: WT**

**
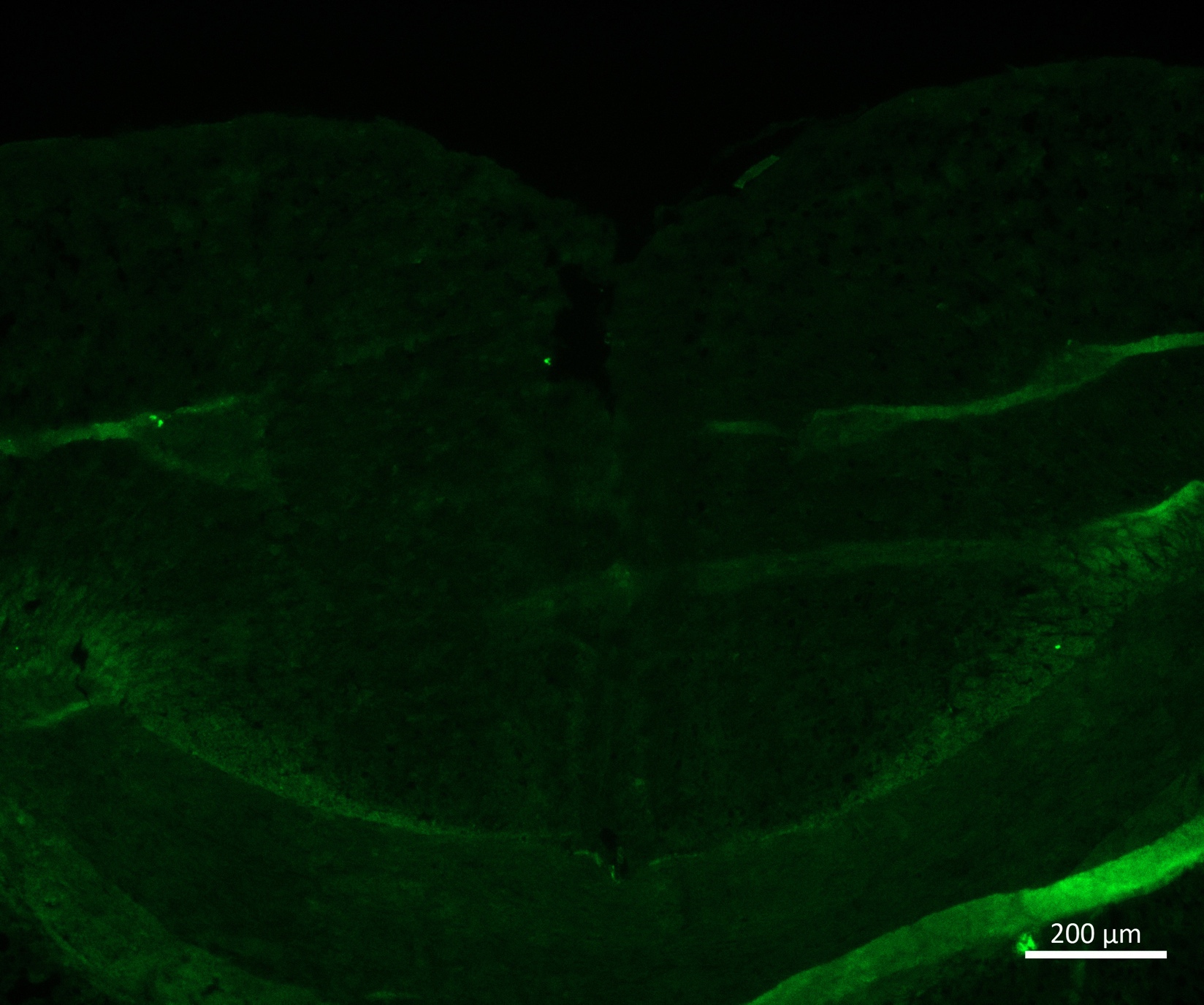
**

**D11: 5XFAD**

**
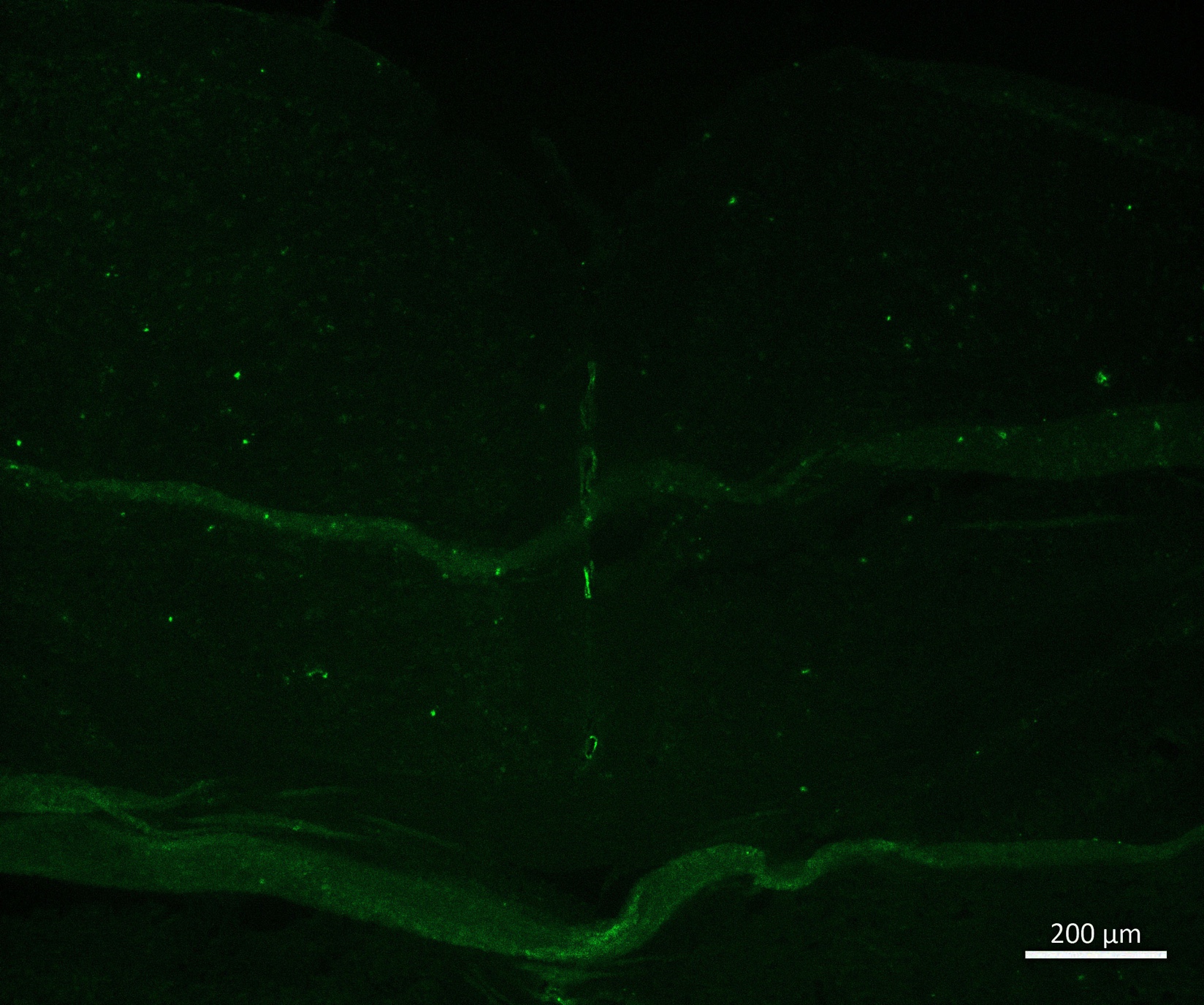
**

**F9: WT**

**
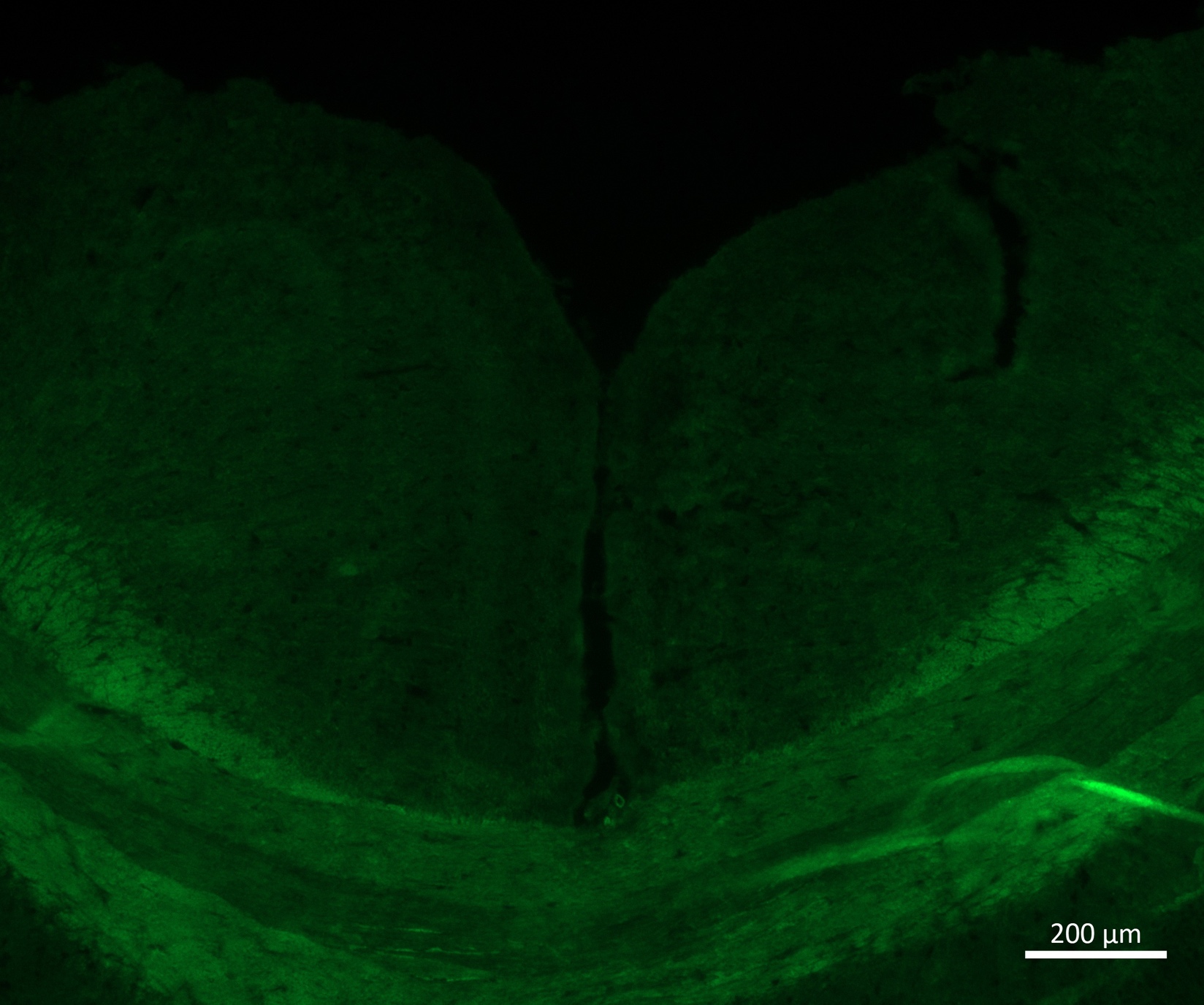
**

**F9: 5XFAD**

**
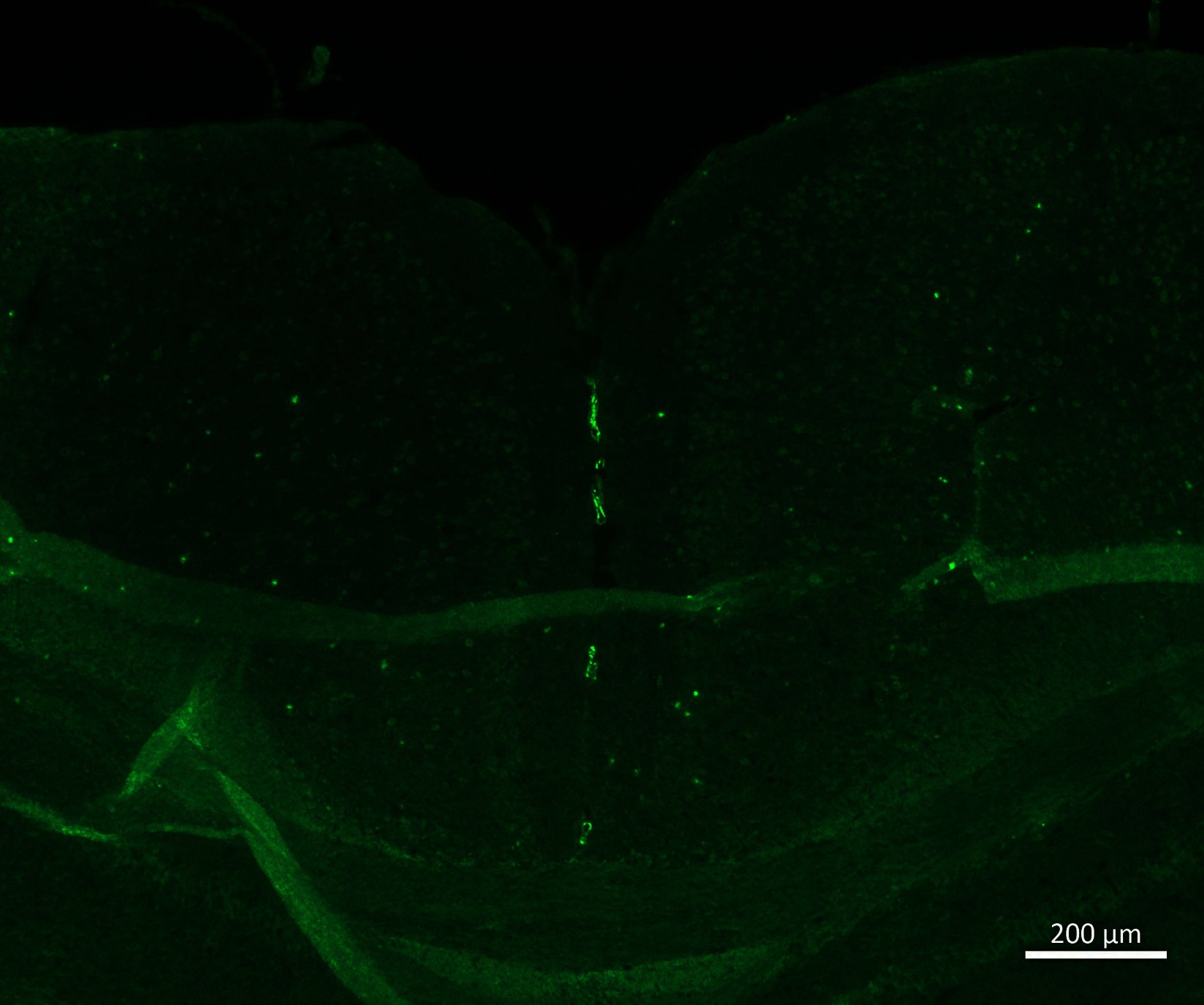
**

**H5: WT**

**
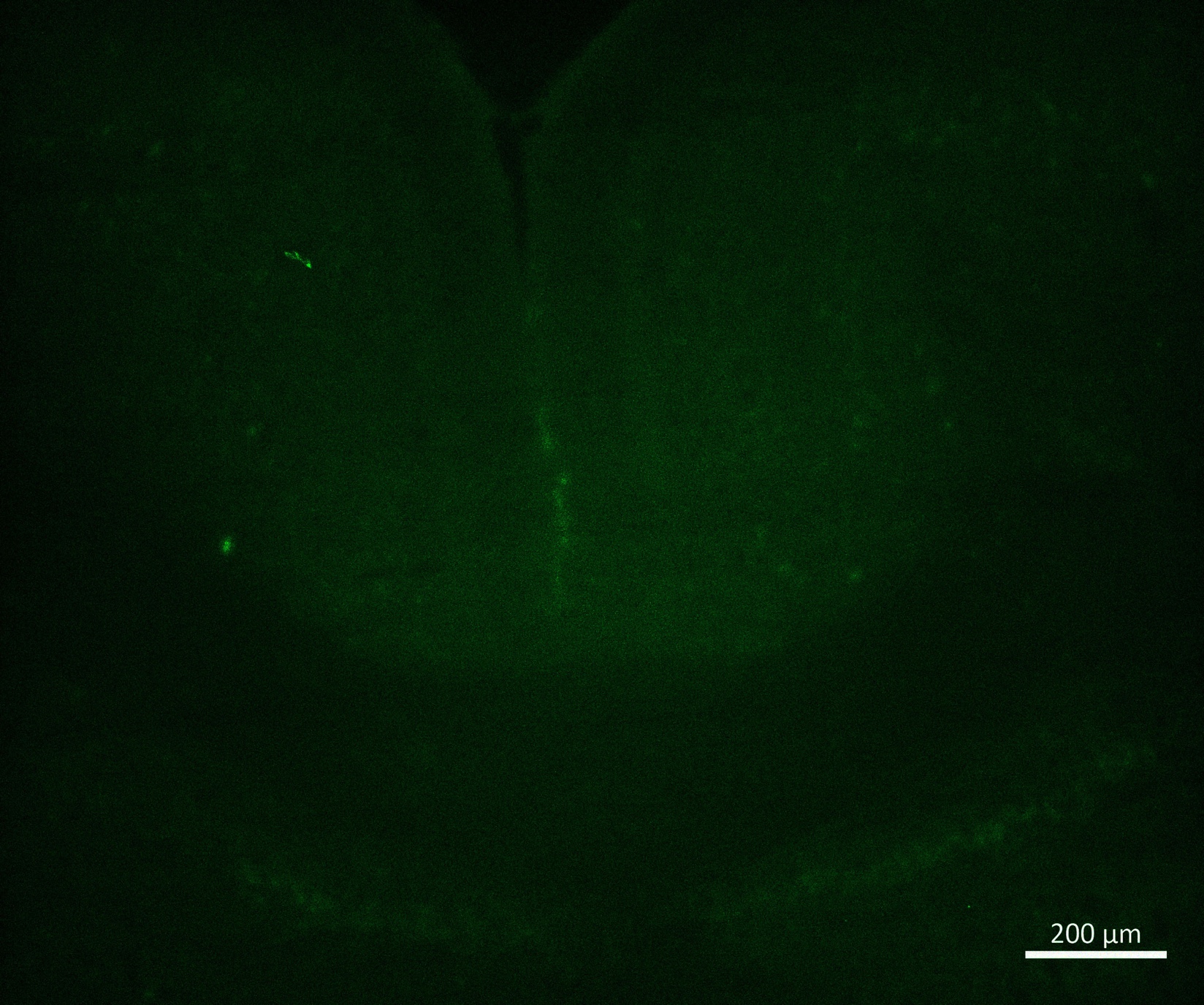
**

**H5: 5XFAD**

**
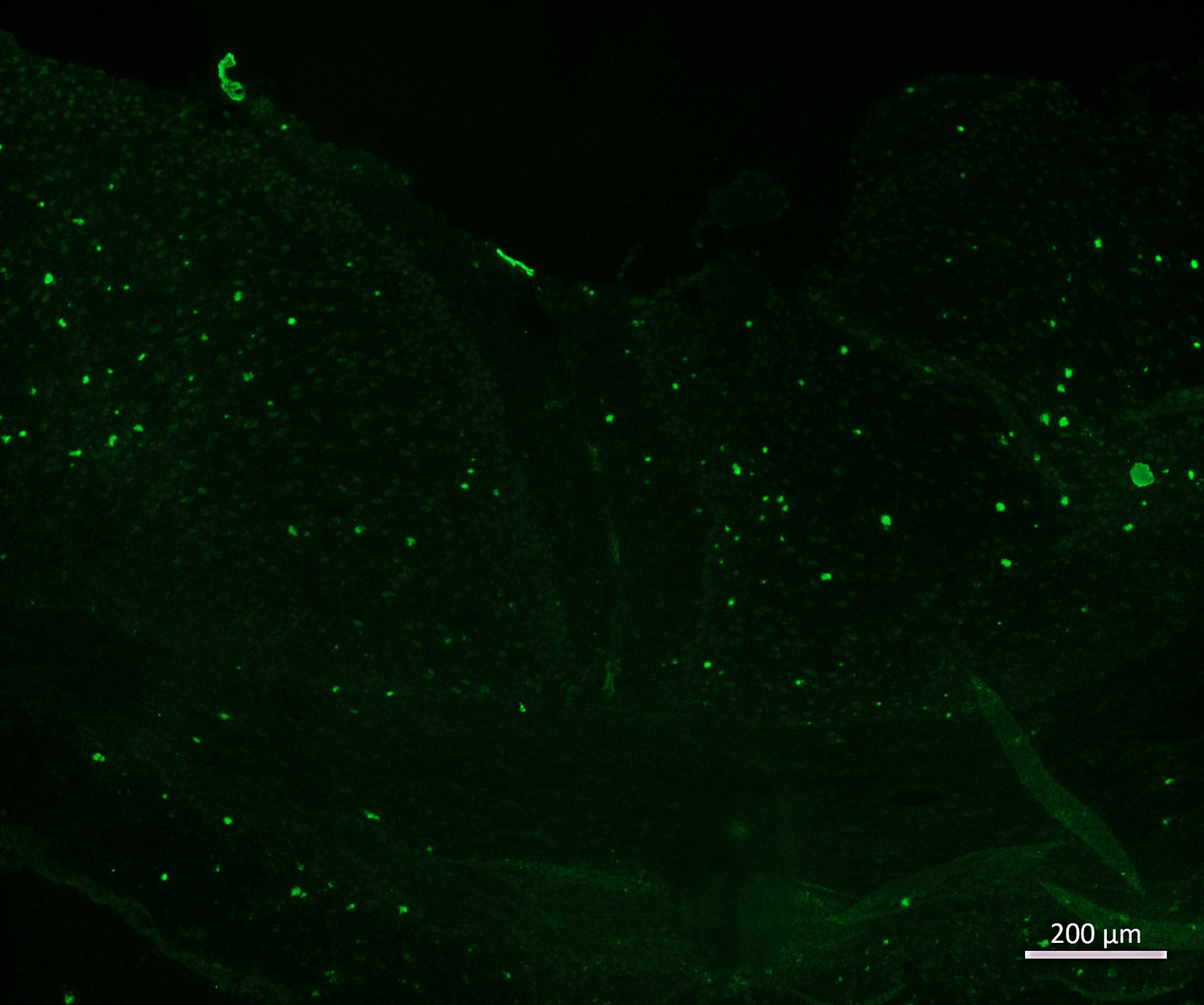
**

**E3: WT**

**
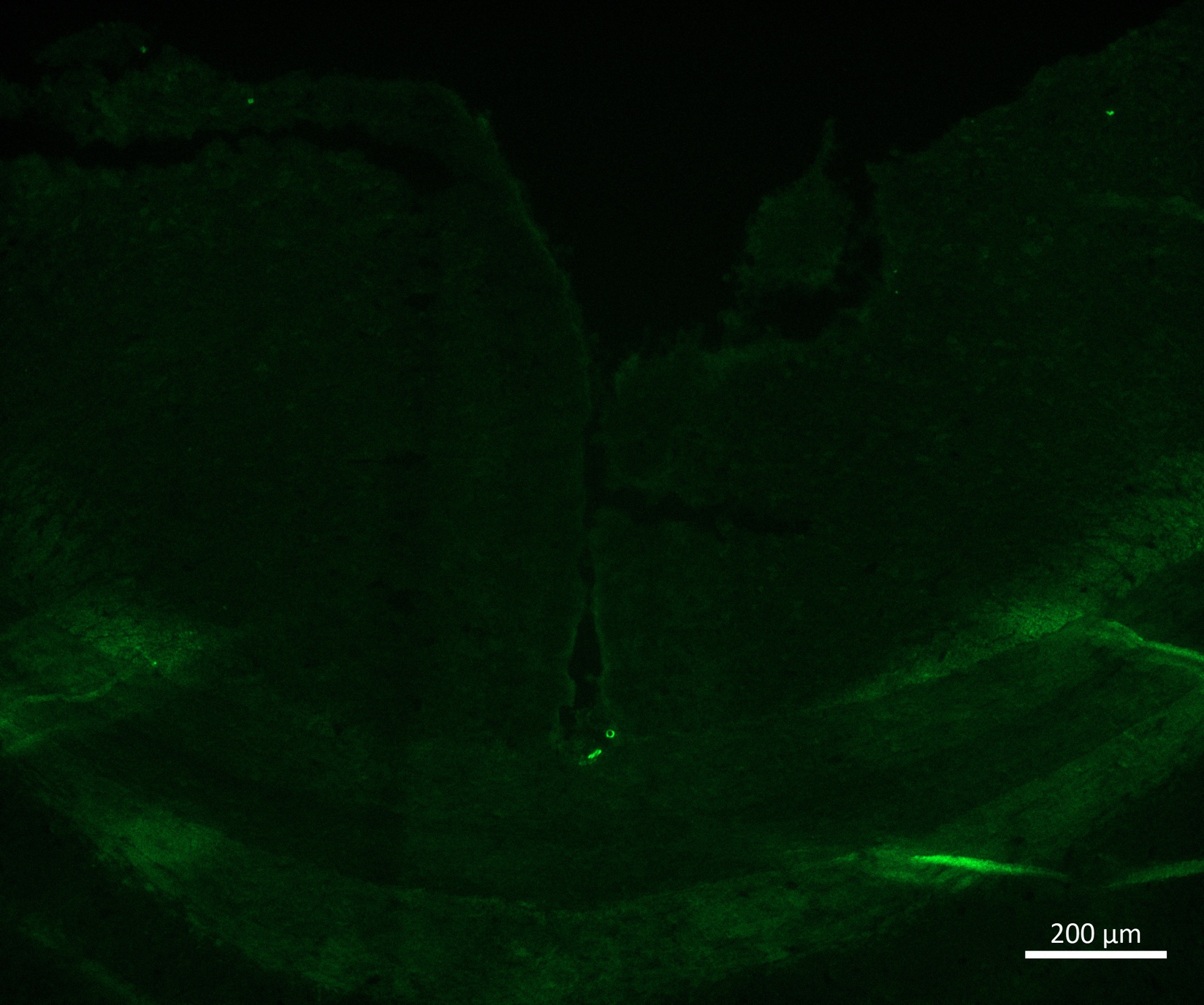
**

**E3: 5XFAD**

**
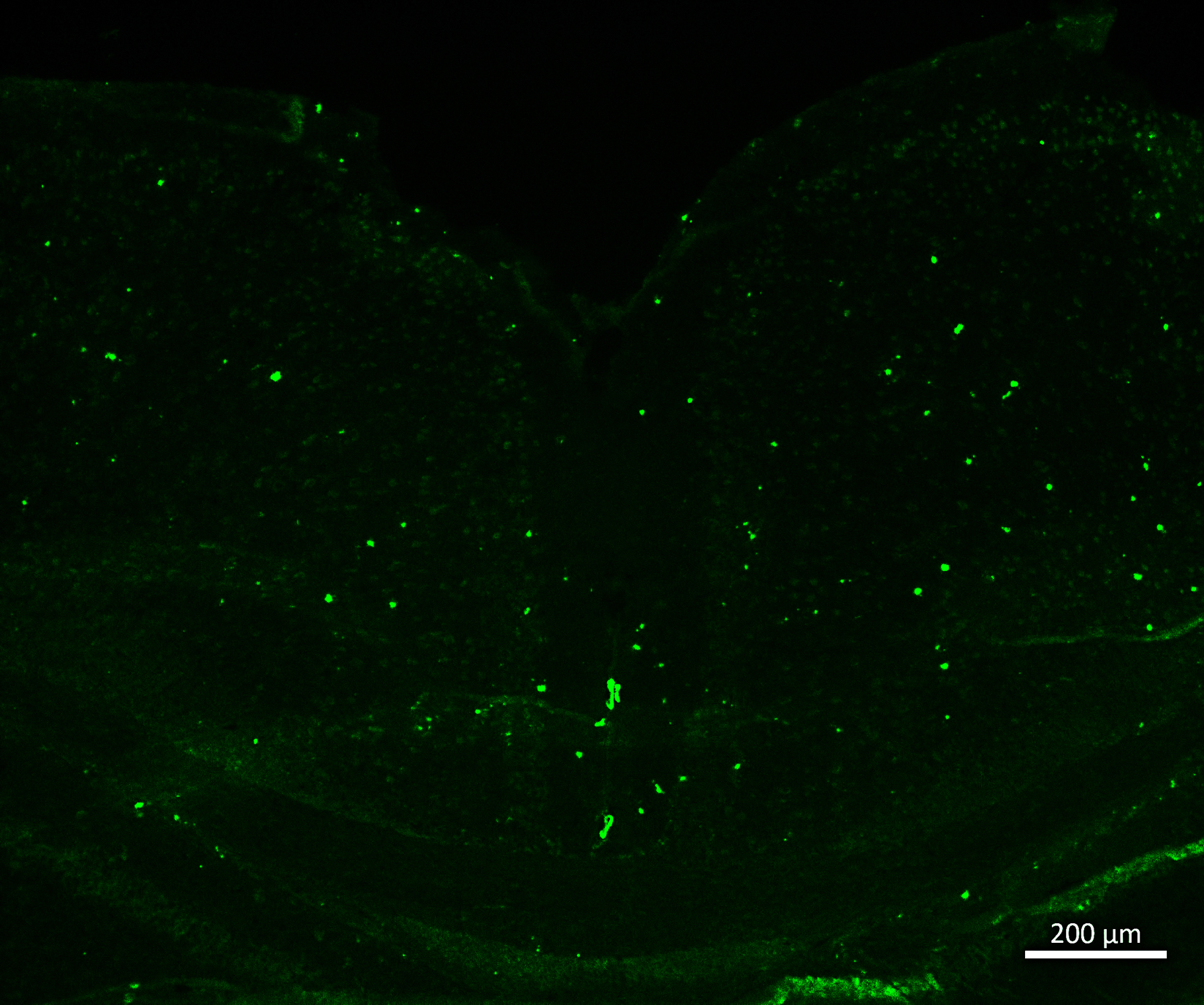
**

**Figure S6**. **Labeled panel of dot blots to screen the specificity of E3 nanobody for different amyloid-β species.** The top blot was stained with 6E10, a commercially available antibody**.** The bottom blot received E3 nanobody as an antibody. Blots were imaged together on a Bio-Rad ChemiDoc MP imaging system using corresponding excitation/emission wavelengths.


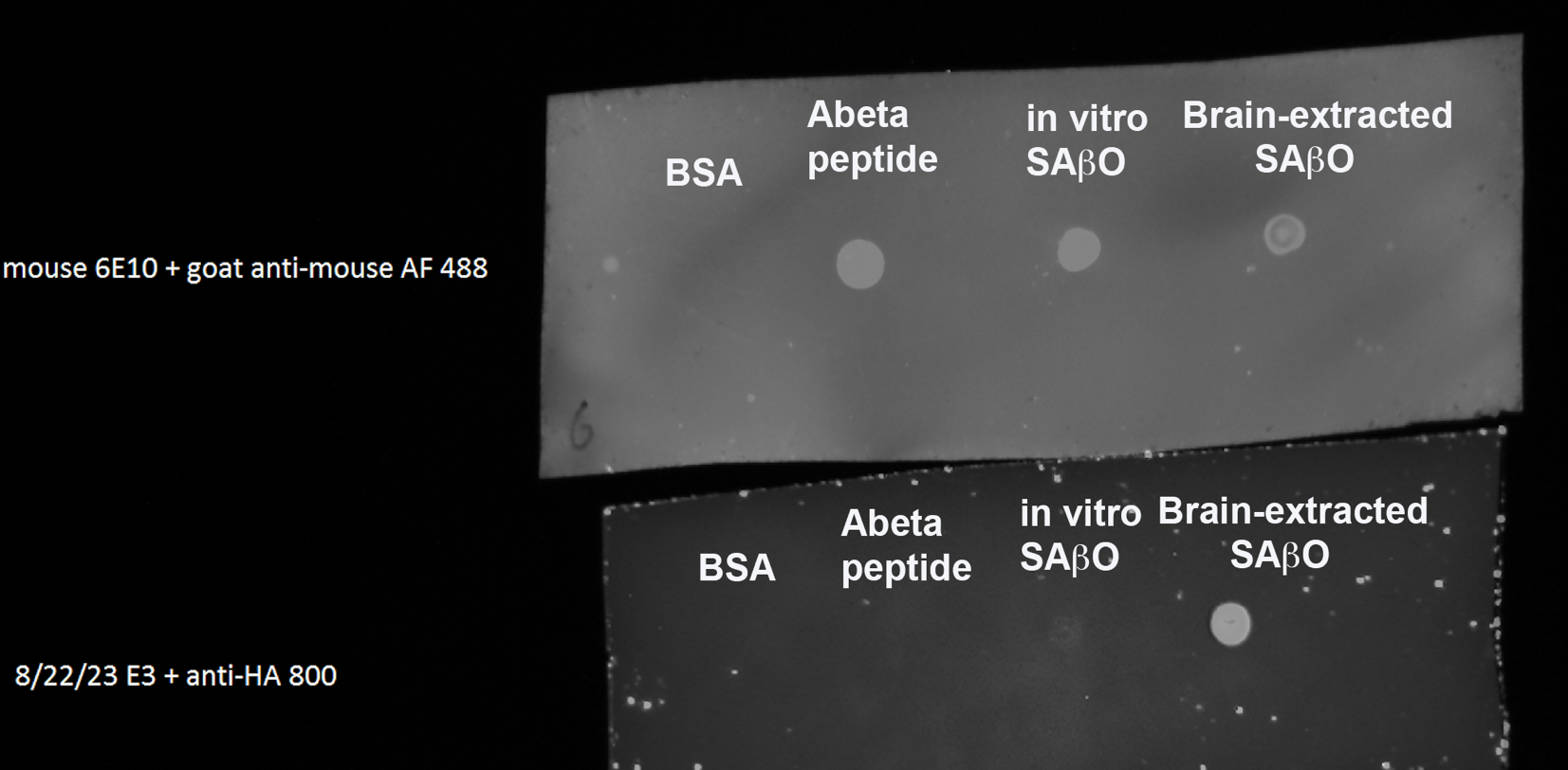


**Figure S7**. (A) Using dot blot analysis as described in **Figure S6**, aside from SAβO, E3 nanobody also recognizes amyloid-β plaques isolated from 5XFAD brains; (B) The presence of amyloid-β plaques in 5XFAD brains was confirmed by 6E10 antibody (arrow).


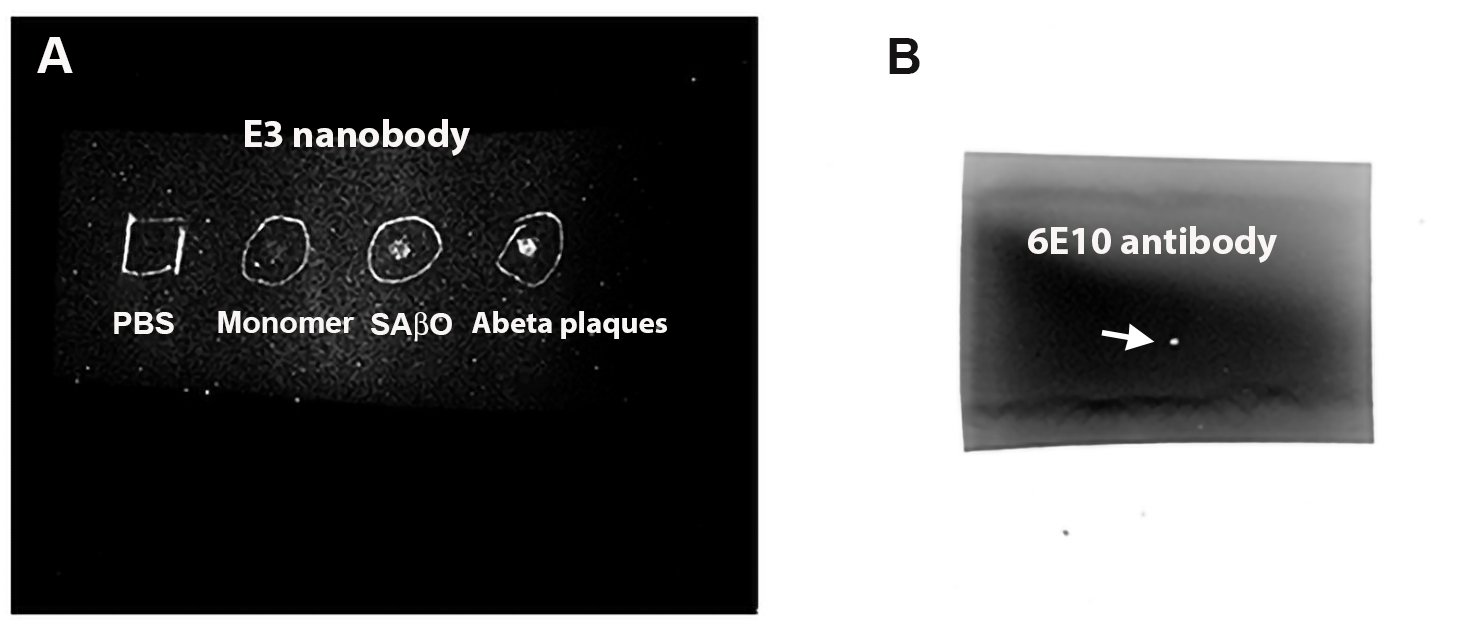


**Figure S8**. AlphaFold suggests potential binding modes. A. A single AlphaFold model of nanobody E3. Complementary Determining Regions (CDRs) are colored green for CDR1, yellow for CDR2, and orange for CDR3. The unusual orientation of CDR3 (relatively flat and jutting away from the nanobody) was present in all 20 models (B) but was associated with relatively low confidence values (C). Panel C shows a color ramp from low to high confidence based on AlphaFold’s estimates, suggesting low confidence in the CDR3 orientation. Panels C and D show E3 positioned next to a structure of a human amyloid-β(1–42) fibril (pdb 5OQV from ^62^). The inter strand spacing of the beta-solenoid structure of the fibril is such that the CDRs of E3 could easily contact multiple Aβ peptides.


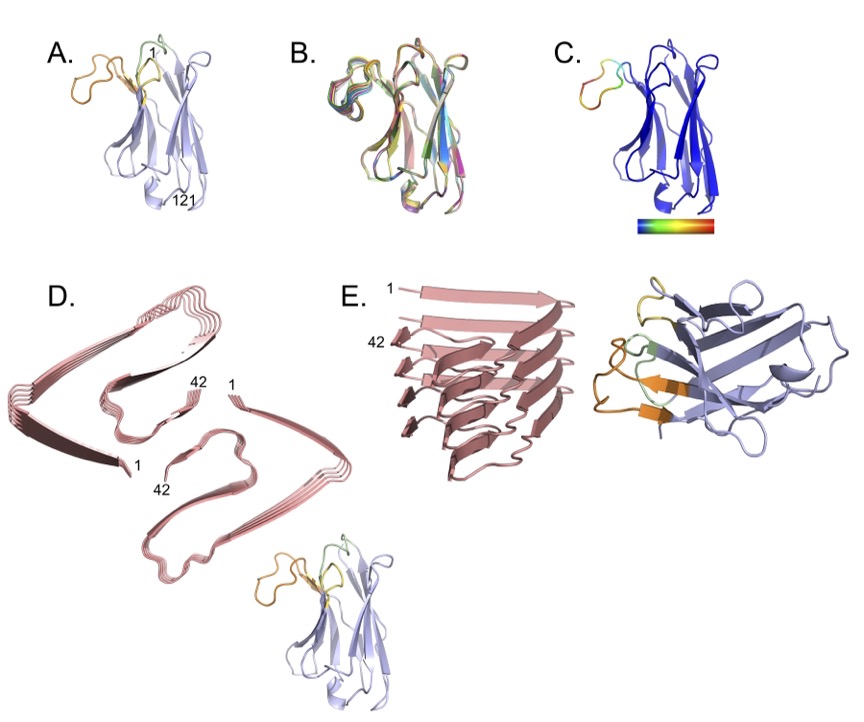

Supplement: Supplementary file 1 — Supplementary Information. [file 41598_2024_66970_MOESM1_ESM.docx]
